# ABCB1 protects bat cells from DNA damage induced by genotoxic compounds

**DOI:** 10.1038/s41467-019-10495-4

**Published:** 2019-06-27

**Authors:** Javier Koh, Yoko Itahana, Ian H. Mendenhall, Dolyce Low, Eunice Xin Yi Soh, Alvin Kunyao Guo, Yok Teng Chionh, Lin-Fa Wang, Koji Itahana

**Affiliations:** 1 Programme in Cancer & Stem Cell Biology, Duke-NUS Medical School, 8 College Road, Singapore, 169857 Singapore; 2 Programme in Emerging Infectious Diseases, Duke-NUS Medical School, 8 College Road, Singapore, 169857 Singapore; 3 School of Applied Science, Temasek Polytechnic, 21 Tampines Avenue 1, Singapore, 529757 Singapore

**Keywords:** Cancer prevention, Transporters, DNA damage response

## Abstract

Bats are unusual mammals, with the ability to fly, and long lifespans. In addition, bats have a low incidence of cancer, but the mechanisms underlying this phenomenon remain elusive. Here we discovered that bat cells are more resistant than human and mouse cells to DNA damage induced by genotoxic drugs. We found that bat cells accumulate less chemical than human and mouse cells, and efficient drug efflux mediated by the ABC transporter ABCB1 underlies this improved response to genotoxic reagents. Inhibition of ABCB1 triggers an accumulation of doxorubicin, DNA damage, and cell death. ABCB1 is expressed at higher levels in several cell lines and tissues derived from bats compared to humans. Furthermore, increased drug efflux and high expression of ABCB1 are conserved across multiple bat species. Our findings suggest that enhanced efflux protects bat cells from DNA damage induced by genotoxic compounds, which may contribute to their low cancer incidence.

## Abstract (web summary)

Bats possess an extended lifespan compared to most mammals of their size, and have a low cancer incidence. Here the authors show that several bat species exhibit resistance to genotoxic agents that is in part attributable to high expression of the ABCB1 transporter.

## Introduction

Cancer is a leading cause of death among aging populations. Current cancer treatments are inefficient and have unacceptable side-effects, due in part to the biological complexity and incomplete understanding of the disease^1^. Therefore, there is an urgent need to develop alternative therapeutics. In-depth understanding of tumour suppressive mechanisms can provide effective strategies to treat and prevent cancer.

Most cancer research utilises cancer-prone, short-lived animals such as mouse and rat, which are amenable to genetic manipulation. Although these model organisms share some tumour suppressor mechanisms with humans, emerging evidence suggests that long-lived mammals have evolved unique and robust anti-cancer strategies^2^. For example, genome and transcriptome analyses of the bowhead whale, a large and long-lived mammal with a low rate of cancer, revealed a positive evolutionary selection of the *excision repair cross-complementation group 1* (*ERCC1*) gene and duplication of the *proliferating cell nuclear antigen* (*PCNA*) gene, both DNA repair genes^3^. These data imply that an enhanced DNA repair pathway might protect whales from cancer by lowering the mutation frequency. Genomic analysis of elephants, which also have a low cancer incidence, uncovered a high number of *TP53* pseudogenes, and elephant cells displayed an enhanced TP53-dependent DNA damage response compared to human cells^4,5^.

Some small mammals also show remarkable cancer resistance. Early contact inhibition is a unique mechanism of tumour suppression in the naked mole rat, mediated by the secretion of high-molecular-mass hyaluronic acid^6,7^. Blind mole rats also exhibit remarkable cancer resistance by inducing concerted necrotic cell death in response to hyperplasia^8^ and by having a stronger extracellular matrix to restrict tumour growth and metastasis^9^. Unravelling the mechanisms underlying low cancer rates provides important perspectives and insights into cancer biology and potential treatment strategies for humans.

Bats are small, long-lived mammals with an extremely low incidence of cancer^2,10^. They are the second largest order of mammals in the world^11^, the only mammal capable of powered-wing flight, and an asymptomatic reservoir for many deadly viruses^10^. Their longevity data predominately come from field-based studies, and therefore, their true longevities may be underestimated, and they may live longer than these reported records^12–14^. In general, longevity is positively correlated with the body size^12,13^. Austad and Fischer^13^ defined the longevity quotient (LQ) which takes the consideration of body mass in the estimated maximum lifespan of individual mammalian species. Bats possess one of the highest LQ value among the mammal order^12,13^, indicating that bats live much longer than other mammals of equivalent size. Their higher LQ makes bats interesting species to study since they may have unique tumour suppressive mechanisms compared to humans. Only a handful of cases of tumours have been recorded to date for bats in captivity^15–17^. However, the underlying mechanisms of tumour suppression in bats are still not fully understood. To understand such mechanisms, we previously performed genomic analyses of *Pteropus alecto* *(P. alecto)* and *Myotis davidii*, and discovered that the DNA damage response (DDR) pathway of bats had undergone positive evolutionary selection^18^. The DDR pathway is the major mechanism that detects and repairs damaged DNA and highly conserved in a diverse range of organisms^19^. DNA damage can induce cancer via multiple mechanisms, including genomic instability, inactivation of tumour suppressors or activation of oncogenes^20^. Living organisms are constantly exposed to environmental xenobiotics and internal metabolic by-products, some of which can possess genotoxic properties that can lead to DNA damage^21,22^. Elucidating whether and how bats efficiently resist DNA damage will advance our understanding of cancer biology.

Here, we describe a role for the ATP-binding cassette (ABC) transporter ABCB1 in DNA damage resistance in bats. We propose genotoxic efflux as a potential tumour suppressive mechanism that contributes to the low incidence of cancer in bats.

## Results

### Bat-derived cells respond distinctively to chemical exposure

To explore DNA damage resistant mechanisms in bat, we first examined the DNA damage response in bat, human and mouse cells exposed to different DNA damaging treatments.

**Fig. 1:**
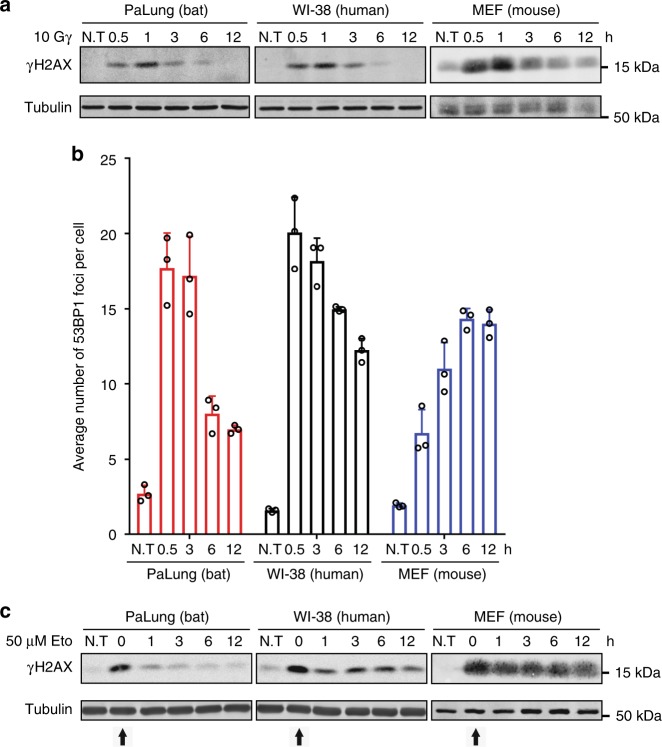
γH2AX and 53BP1 responses to γ-irradiation and etoposide in bat, human and mouse cells. **a** Western blot analysis of γH2AX in PaLung, WI-38 and MEF cells exposed to 10 Gy of γ-irradiation. Protein lysates were harvested at the indicated time points. Tubulin was used as a loading control. **b** Analysis of the average number of 53BP1 foci per cell for PaLung, WI-38 and MEF cells treated with 10 Gy of γ-irradiation. Immunofluorescence staining of 53BP1 was performed at the indicated time points. The number of foci in a minimum of 100 cells was quantified. Bars represent the means ± SDs of three independent experiments. **c** Western blot analysis of γH2AX in PaLung, WI-38 and MEF cells treated with 50 μM etoposide (Eto) for 3 h, followed by drug-free medium up to 12 h (starting at *t* = 0 h, indicated by arrow). Protein lysates were harvested at the indicated time points. Tubulin was used as a loading control. N.T stands for no treatment. All Western blot results shown are representative of at least three experimental repeats

First, we exposed PaLung (normal lung fibroblast from *P. alecto*), WI-38 (human normal lung fibroblast) and MEF (mouse embryonic fibroblast) cells to γ-irradiation, which induces double-strand breaks (DSBs) in DNA via free radical formation^23^. We exposed cells to 10 Gy of γ-irradiation and monitored the well-established DNA DSB marker phosphorylated histone 2A variant X (γH2AX)^24^. We found that γH2AX accumulated and diminished with similar kinetics in PaLung, WI-38 and MEF cells exposed to γ-irradiation (Fig. 1a). *P. alecto* PaKiT03 cells (kidney cells transformed with SV40 large T antigen) and human HEK293T cells (embryonic kidney cell transformed with SV40 large T antigen) also showed similar changes in γH2AX levels in response to γ-irradiation (Supplementary Fig. 1A).

To verify that elevated γH2AX represented a DSB response rather than a preapoptotic signal associated with high levels of pan-nuclear γH2AX^25^, we monitored γH2AX in cells by immunofluorescence. We found that cells exposed to γ-irradiation displayed γH2AX foci rather than pan-nuclear accumulation (Supplementary Fig. 1B), consistent with DSBs. To further confirm these results, we detected the DNA repair protein p53-binding protein 1 (53BP1) by immunofluorescence. 53BP1 is recruited to DSBs to facilitate non-homologous end-joining repair and is released upon repair^26,27^. We observed an increase in the number of 53BP1 foci in PaLung and WI-38 cells exposed to γ-irradiation, which reduced gradually and similarly over time (Fig. 1b). On the other hand, the number of 53BP1 foci increased slowly in γ-irradiated MEFs and remained high at least 12 h after irradiation (Fig. 1b), consistent with a previous report about DNA repair^28^. Together, these results suggest that lung fibroblasts from *P. alecto* and human are similarly sensitive and responsive to DNA damage induced by ionising radiation, whereas MEFs display a slightly slower response to the same treatment.

Next, we treated the same set of cell lines with the chemotherapeutic drug etoposide (50 µM). Etoposide inhibits topoisomerase II^29^ and thus induces DNA DSBs. We treated cells for 3 h, washed away the drug, and monitored the levels of γH2AX over time after drug removal. γH2AX was similarly induced by etoposide in all three cell lines (Fig. 1c, at 0 h time point after treatment). Unexpectedly, γH2AX levels returned to almost basal levels within 1–3 h of etoposide removal in PaLung cells, whereas it remained elevated for at least 12 h in WI-38 and MEF cells (Fig. 1c). Similarly, γH2AX levels returned to basal levels within 3 h of etoposide removal in *P. alecto* PaKiT03 cells, but remained high in human HEK293T cells (Supplementary Fig. 1C). These results suggest that *P. alecto* cells are more resistant than human and mouse cells to etoposide-induced DNA damage.

### Bat cells accumulate less doxorubicin than human and mouse cells

**Fig. 2:**
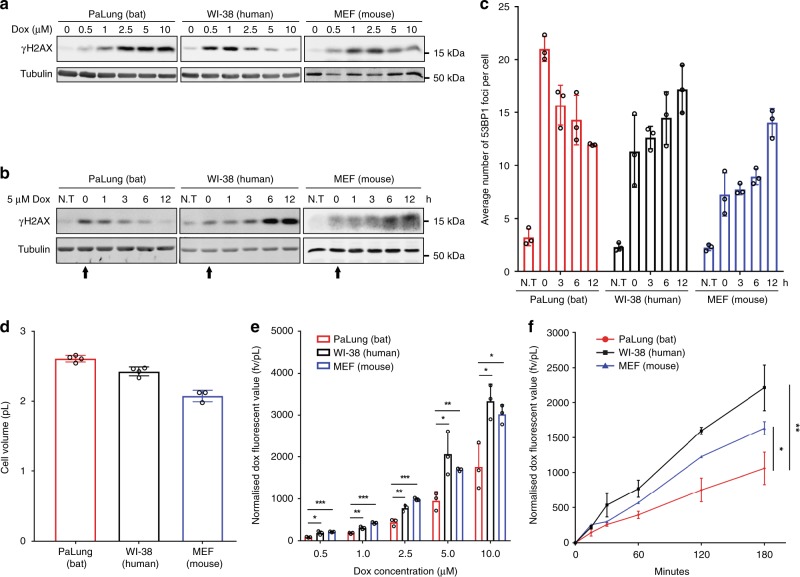
Analysis of γH2AX, 53BP1, and intracellular accumulation of doxorubicin in bat, human, and mouse cells. **a** Dose titration of doxorubicin (Dox) in PaLung, WI-38 and MEF cells. Cells were treated with the indicated doses of doxorubicin for 3 h, and protein lysates were analysed by Western blotting. Tubulin was used as a loading control. **b** Western blot analysis of γH2AX in PaLung, WI-38 and MEF cells treated with 5 μM Dox for 3 h, followed by drug-free medium up to 12 h (starting at *t* = 0 h, indicated by arrow). Protein lysates were harvested at the indicated time points. Tubulin was used as a loading control. N.T stands for no treatment. **c** Analysis of the average number of 53BP1 foci per cell for PaLung, WI-38 and MEF cells. Cells were treated with 5 μM Dox for 3 h, followed by drug-free medium up to 12 h. Immunofluorescence staining of 53BP1 was performed at the indicated time points. The number of foci in a minimum of 100 cells was quantified. N.T stands for no treatment. **d** The volume of the indicated cell lines. **e** Doxorubicin accumulation in PaLung, WI-38 and MEF cells after 3 h of incubation at the indicated concentration. The amount of accumulated doxorubicin in cells was analysed by flow cytometry. The mean fluorescence value (fv) of doxorubicin is normalised to cell volume (pL). **f** Time course of doxorubicin accumulation in PaLung, WI-38, and MEF cells. Cells were treated with 5 μM doxorubicin for the indicated time and analysed by flow cytometry. The mean fluorescence value of doxorubicin accumulated in each cell line is normalised to cell volume. All Western blot results shown are representative of at least three experimental repeats. Bars represent the means ± SDs of at least three independent experiments. Statistical significances were calculated using unpaired student’s two-sided *t* test. *p* < 0.05 is represented with *, *p* < 0.01 with ** and *p* < 0.001 with ***

To extend our observations, we evaluated the response of these cells to the chemotherapy reagent doxorubicin. Like etoposide, doxorubicin inhibits topoisomerase II and induces DNA DSBs that lead to γH2AX induction. However, high concentrations of doxorubicin can cause histone eviction that leads to reduced levels of γH2AX in human cells^30^. Indeed, WI-38 cells treated for 3 h with increasing concentrations of doxorubicin showed a dose-dependent decrease in γH2AX levels (Fig. 2a), consistent with histone eviction^30^. MEFs displayed a similar dose-dependent decrease in γH2AX levels (Fig. 2a). On the other hand, PaLung cells displayed a dose-dependent increase in γH2AX levels (Fig. 2a), consistent with increased DNA damage but without histone eviction.

As we did with etoposide, we treated cells with 5 µM doxorubicin for 3 h, washed away the drug, and monitored γH2AX levels over time for up to 12 h. We found that γH2AX in PaLung cells started to decrease immediately after washout and returned to almost basal levels within 6 h (Fig. 2b). In contrast, γH2AX levels in WI-38 and MEF cells accumulated after washout and were sustained at high levels until the last time point we monitored (Fig. 2b). The accumulation of γH2AX after doxorubicin washout was similarly observed in various human cell lines, but not in various *P. alecto* cell lines (Supplementary Fig. 2A).

To monitor DSB formation, we again detected γH2AX by immunofluorescence. We found that doxorubicin-treated PaLung, WI-38, and MEF cells displayed γH2AX foci but not pan-nuclear staining, consistent with DSBs (Supplementary Fig. 2B). Similar to γH2AX levels, 53BP1 foci in PaLung cells diminished soon after removal of 5 µM of doxorubicin (Fig. 2c). In contrast, 53BP1 foci accumulated in WI-38 and MEF cells after removal of doxorubicin (Fig. 2c).

Together, these findings suggest that treatment with 5 µM doxorubicin does not lead to histone eviction in *P. alecto* cells. In contrast, similarly treated human and mouse cells display transient histone eviction; the accumulation of γH2AX in human and mouse cells over time might reflect a decrease in intracellular doxorubicin levels, such that there is DNA damage without histone eviction.

We hypothesised that *P. alecto* cells accumulate less doxorubicin than similarly treated human and mouse cells, which would protect them from histone eviction since the only high dose of doxorubicin is known to cause histone eviction^30^ (Fig. 2a). To test this hypothesis, we treated the cells with increasing doses of doxorubicin and measured intracellular doxorubicin accumulation by flow cytometry^31^. The amount of intracellular doxorubicin was normalised to the cellular volume since there was some variation of the cell size among the cell lines (Fig. 2d). We found that the amount of doxorubicin within PaLung cells was significantly lower than that within WI-38 and MEF cells, at all the treatment concentrations (Fig. 2e) and times (Fig. 2f). Similar trends were observed in *P. alecto* PaKiT03 cells in comparison with human HEK293T cells (Supplementary Fig. 2C–E). Together, these results suggest that *P. alecto* cells are more resistant than human and mouse cells to doxorubicin-induced DNA damage at least in part due to reduced accumulation of doxorubicin.

### Bat cells exhibit efficient drug efflux via ABC transporters

**Fig. 3:**
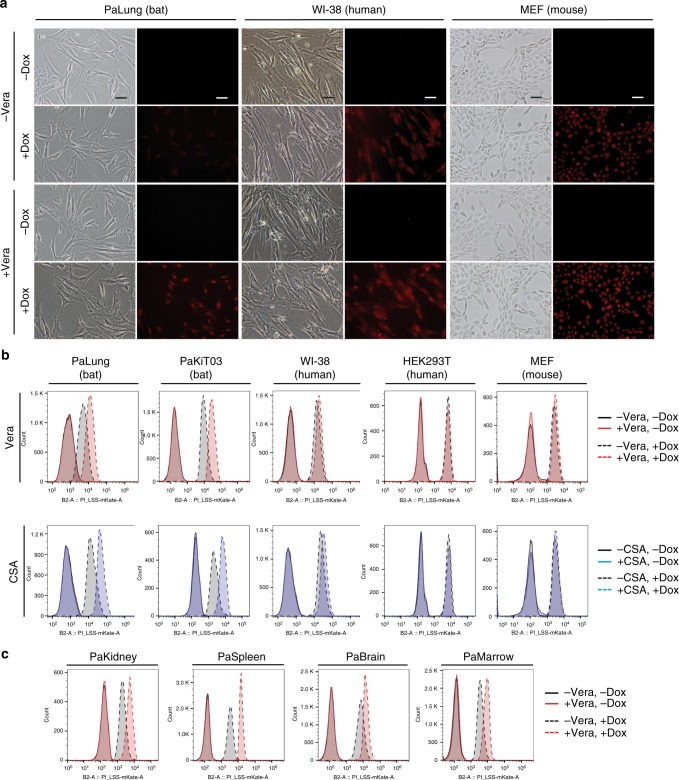
Analysis of drug efflux capability via ABC transporters. **a** Doxorubicin (Dox) fluorescence or phase contrast images of PaLung, WI-38, and MEF cells. Cells were pre-treated without or with 5 μM verapamil (Vera) for 30 min before the treatment with 10 μM doxorubicin alone or together with Vera for an additional 3 h. Images were acquired with 20× objective lens, and scale bar represents 50 μm. **b** Flow cytometry analyses of Dox accumulation in PaLung, PaKiT03, WI-38, HEK293T, and MEF cells. Cells were pre-treated without or with 5 μM verapamil (Vera, top row) or 5 μM cyclosporin A (CSA, bottom row) for 30 min before the treatment with 10 μM doxorubicin alone or together with Vera or CSA for an additional 3 h. **c** Flow cytometry analyses of Dox accumulation in cells derived from different tissues of *P. alecto* in the presence or absence of verapamil. Cells were treated and analysed as in (**b**). All results shown are representative of at least three experimental repeats

Intracellular drug concentrations are influenced by the balance of drug influx and efflux. ABC transporters are the primary transporters that efflux genotoxic substances, thereby reducing intracellular xenobiotic concentrations^32^. To determine if ABC transporters contribute to the difference in doxorubicin accumulation between bat, human and mouse cells, we used verapamil (Vera) to inhibit efflux via ABC transporters^32^, and assessed doxorubicin accumulation by fluorescence microscopy and flow cytometry. Specifically, we pre-treated cells with or without verapamil for 30 min, added doxorubicin for 3 h and measured intracellular doxorubicin levels. No notable differences were observed in the doxorubicin fluorescence intensities between control and verapamil-pre-treated WI-38 cells (Fig. 3a, b), suggesting that WI-38 cells exhibit no endogenous drug efflux capability via ABC transporters. Similarly, no obvious differences were detected in doxorubicin accumulation between control and verapamil-pre-treated MEF cells (Fig. 3a, b) and HEK293T cells (Fig. 3b and Supplementary Fig. 3). In contrast, verapamil-pre-treatment increased doxorubicin fluorescence in PaLung and PaKiT03 cells (Fig. 3a, b and Supplementary Fig. 3). Likewise, pre-treatment with cyclosporin A (CSA), another ABC transporter inhibitor^32^, also led to an increase in doxorubicin fluorescence in PaLung and PaKiT03 cells, but not in WI-38, HEK293T, and MEF cells (Fig. 3b). These results suggest that the drug efflux activity of ABC transporters is required to reduce doxorubicin accumulation in PaLung and PaKiT03 cells. Furthermore, primary *P. alecto* cell lines derived from kidney (PaKidney), spleen (PaSpleen), brain (PaBrain), and bone marrow (PaMarrow) also displayed a similar increase in doxorubicin accumulation with verapamil pre-treatment (Fig. 3c). Together, these results suggest that cells derived from multiple tissues of *P. alecto* possess drug efflux activity via ABC transporters.

### Drug efflux in bat cells reduces the exposure to doxorubicin genotoxicity

**Fig. 4:**
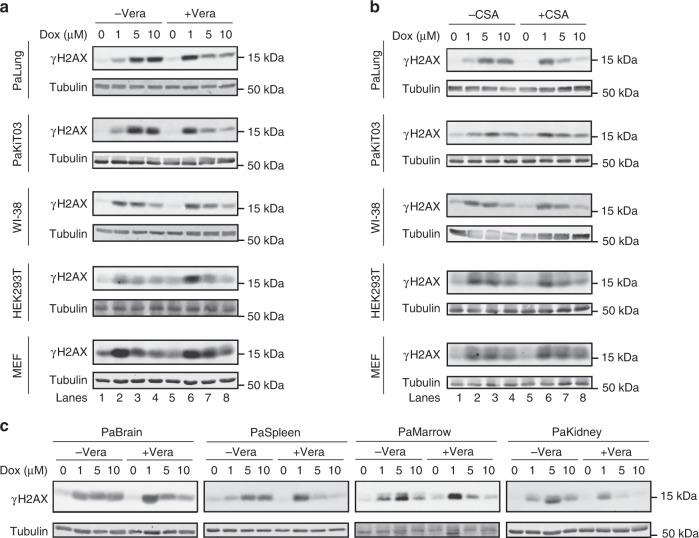
Effect of ABC transporter inhibition on γH2AX induced by doxorubicin. **a**, **b** Western blot analyses of γH2AX induced by increasing doses of doxorubicin (Dox). PaLung, PaKiT03, WI-38, HEK293T and MEF cells were pre-treated without or with 5 µM verapamil (Vera) (**a**) or 5 µM cyclosporin A (CSA) (**b**) for 30 min before the treatment with the increasing amount of DOX alone or together with Vera or CSA for additional 3 h. **c** Cells derived from different tissues of *P. alecto* were treated as in (**a**) and analysed by Western blotting. All results are representative of at least three experimental repeats. Tubulin was used as a loading control

We hypothesised that ABC transporter activity underlies the increased resistance of bat cells to chemotherapeutic drugs. This hypothesis predicts that pre-treatment with inhibitors of ABC transporters will increase the genotoxic effects of doxorubicin in *P. alecto* cells but not in WI-38, HEK293T and MEF cells, which lack substantial ABC transporter activity. Indeed, verapamil pre-treatment enhanced the induction of γH2AX levels by low dose of doxorubicin (1 µM) in PaLung and PaKiT03 cells, but not in WI-38, HEK293T and MEF cells (Fig. 4a, lanes 1, 2, 5 and 6), confirming that the ABC transporter activity helps to prevent DNA damage in PaLung and PaKiT03 cells. Treating PaLung and PaKiT03 cells with higher doses (5 and 10 µM) of doxorubicin in the presence of verapamil led to a doxorubicin dose-dependent decrease in γH2AX levels (Fig. 4a, lanes 6–8), as observed in WI-38, HEK293T and MEF cells with or without verapamil pre-treatment, and consistent with histone eviction^30^ (Fig. 4a, lanes 2–4 and 6–8). Similar results were obtained with the ABC transporter inhibitor CSA (Fig. 4b) and with cells derived from different tissues of *P. alecto* (Fig. 4c). Together, these results demonstrate that broad and efficient drug efflux in *P. alecto* cells contributes to reducing doxorubicin-mediated genotoxicity.

### ABCB1 is responsible for increased drug efflux in bat cells

**Fig. 5:**
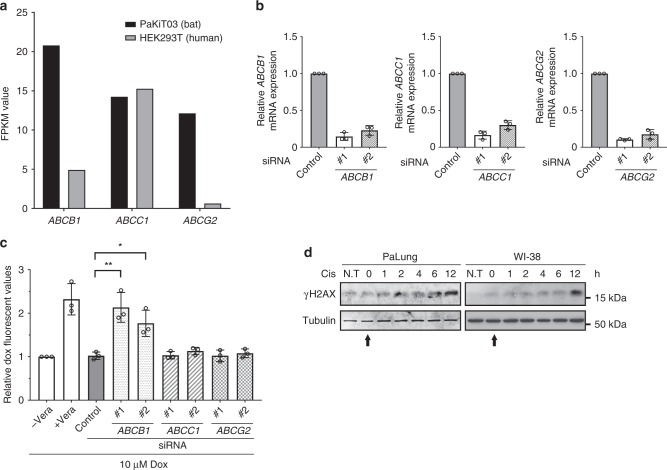
Identification of ABCB1 for the intrinsic doxorubicin efflux capability in bat cells. **a** Relative mRNA expression levels of *ABCB1*, *ABCC1* and *ABCG2* in human HEK293T and *P. alecto* kidney immortalised (PaKiT03) cell lines are shown by FPKM values. **b** qRT-PCR to determine the knockdown efficiency of each ABC transporter. PaKiT03 cells were transfected with control or two independent siRNAs of the indicated genes. mRNA expression of each gene is normalised to *GAPDH* mRNA expression, and presented relative to the control siRNA sample (mean ± SD from three experiments). **c** Doxorubicin (Dox) accumulation in PaKiT03 cells after siRNA knockdown of *ABCB1*, *ABCC1* and *ABCG2*. Cells were treated with 10 µM doxorubicin for 3 h and analysed by flow cytometry. Verapamil (Vera) was used as a positive control for ABC transporter inhibition. Control represents the cells transfected with control siRNA. “Relative values” represents fluorescence intensities of the indicated condition relative to the fluorescent intensity of cells treated Dox alone (“−Vera”, the first bar) (mean ± SD from three experiments). Statistical significances were calculated using unpaired student’s two-sided *t* test. *p* < 0.05 is represented with * and *p* < 0.01 with **. **d** Western blot analysis of γH2AX in PaLung and WI-38 cells at different time points after a 3-h treatment with 10 µg/ml cisplatin (Cis). N.T stands for no treatment (starting at *t* = 0 h, indicated by arrow). The results are representative of three independent experiments. Tubulin was used as a loading control

To determine the transporter(s) responsible for the efflux of doxorubicin in bat cells, we re-analysed our previously published genome-wide transcriptome data set of *P. alecto* kidney cell line PaKiT03^33^. We identified three highly expressed ABC transporters (ABCB1, ABCC1 and ABCG2) that are well-characterised for doxorubicin efflux (Fig. 5a)^32,34^. To determine if one of these transporters is required for doxorubicin efflux in *P. alecto* cells, we reduced the expression of each gene in PaKiT03 cells via small-interfering RNA (siRNA)-mediated knockdown (Fig. 5b) and examined the effects on intracellular doxorubicin accumulation by flow cytometry (Fig. 5c). Verapamil was used as a positive control for efflux inhibition. We found that only *ABCB1* knockdown increased doxorubicin accumulation to a similar extent as verapamil pre-treatment, suggesting that ABCB1 is the transporter responsible for doxorubicin efflux in *P. alecto*-derived cells.

To further verify our observation, we treated WI-38 and PaLung cells with cisplatin, which is not an efflux substrate of ABCB1^35,36^, and assessed γH2AX levels over time after drug removal. We found that WI-38 and PaLung cells treated with cisplatin displayed similar γH2AX induction (Fig. 5d), suggesting that both WI-38 and PaLung cells were equally exposed to the genotoxicity of cisplatin. In contrast, γH2AX induction by etoposide, which is an efflux substrate of ABCB1^34^, resolved earlier in PaLung cells than in WI-38 and MEF cells (Fig. 1c), suggesting that ABCB1-mediated efflux of etoposide reduces its genotoxicity in PaLung cells.

### ABCB1 protects bat cells from DNA damage and cell death

**Fig. 6:**
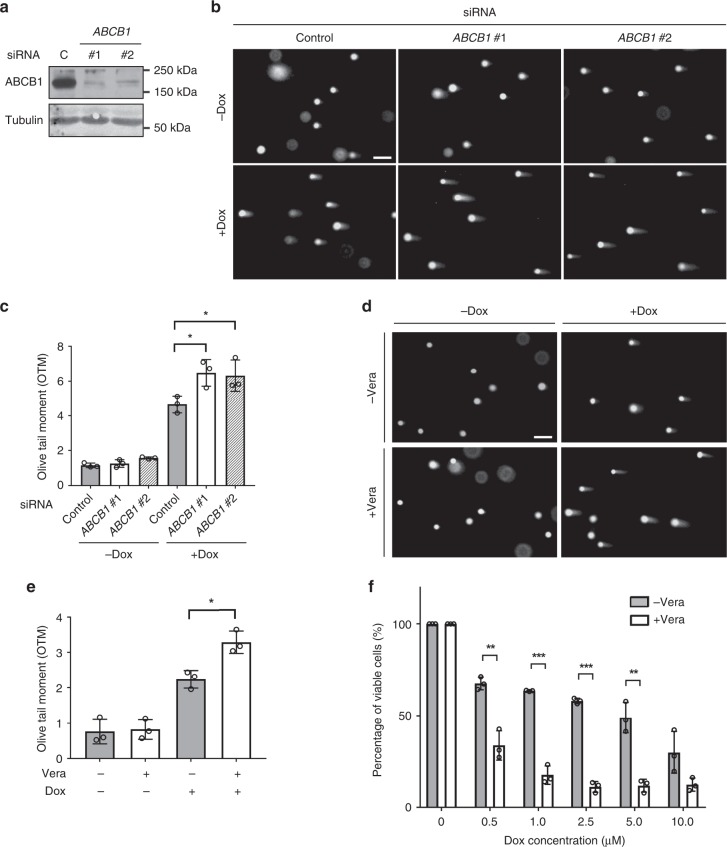
Effect of *ABCB1* knockdown on doxorubicin-induced DNA damage in bat cells. **a** The efficiency of siRNA knockdown. PaKiT03 cells were transfected with the indicated siRNA, and the expression of ABCB1 was analysed by Western blotting. C; control siRNA. Tubulin was used as a loading control. **b** Comet assay of PaKiT03 cells. siRNA knockdown cells were treated without or with 10 µM doxorubicin (Dox) for 3 h, and DNA damage was visualised by comet assay. **c** Quantification of the amount of DNA damage calculated by olive tail moments (OTM) for the cells in (**b**). **d** Comet assay of PaKiT03 cells. Cells were pre-treated without or with 5 µM verapamil (Vera) for 30 min before the treatment with 10 µM doxorubicin alone or together with Vera for an additional 3 h. DNA damage was visualised by comet assay. **e** Quantification of the amount of DNA damage calculated by OTM for the cells in (**d**). **f** Quantification of cell viability. Cells were pre-treated without or with 5 µM verapamil for 30 min before the treatment with the indicated amount of doxorubicin alone or together with verapamil for an additional 3 h. Doxorubicin was then removed and replaced with the doxorubicin-free medium in the absence or presence of verapamil for 72 h before measuring cell viability. Cell viability (%) is presented relative to the control cells (the first grey bar). All data shown have at least three experimental repeats and the images are representative. Scale bar represents 50 µm. Bars represent the means ± SDs of three independent experiments. Statistical significances were calculated using unpaired student’s two-sided *t* test. *p* < 0.05 is represented with *, *p* < 0.01 with ** and *p* < 0.001 with ***

To assess whether ABCB1 protects *P. alecto* cells from DNA damage, we used comet assay to directly measure doxorubicin-induced DNA breaks in PaKiT03 cells, with or without siRNA-mediated depletion of *ABCB1* (Fig. 6a). Knockdown of *ABCB1* significantly increased the DNA damage caused by doxorubicin, as shown by the length of comet tails and the quantified olive tail moments (Fig. 6b, c). Consistent with these observations, verapamil pre-treatment of PaKiT03 cells also increased the DNA damage caused by doxorubicin (Fig. 6d, e), supporting the conclusion that ABCB1 helps to prevent the DNA damage induced by doxorubicin in *P. alecto* cells.

In addition, we evaluated the effect of ABCB1 inhibition on the viability of *P. alecto* cells treated with doxorubicin. We cultured PaKiT03 cells in media (+ or − verapamil) with doxorubicin for 3 h, followed by doxorubicin-free media for an additional 72 h. We then assessed cell viability by measuring ATP levels. We found that ABCB1 inhibition significantly reduced the viability of PaKiT03 cells exposed to doxorubicin, further suggesting that ABCB1 transport activity protects cells from genotoxic stress (Fig. 6f).

### ABCB1 is highly and broadly expressed in *P. alecto*

**Fig. 7:**
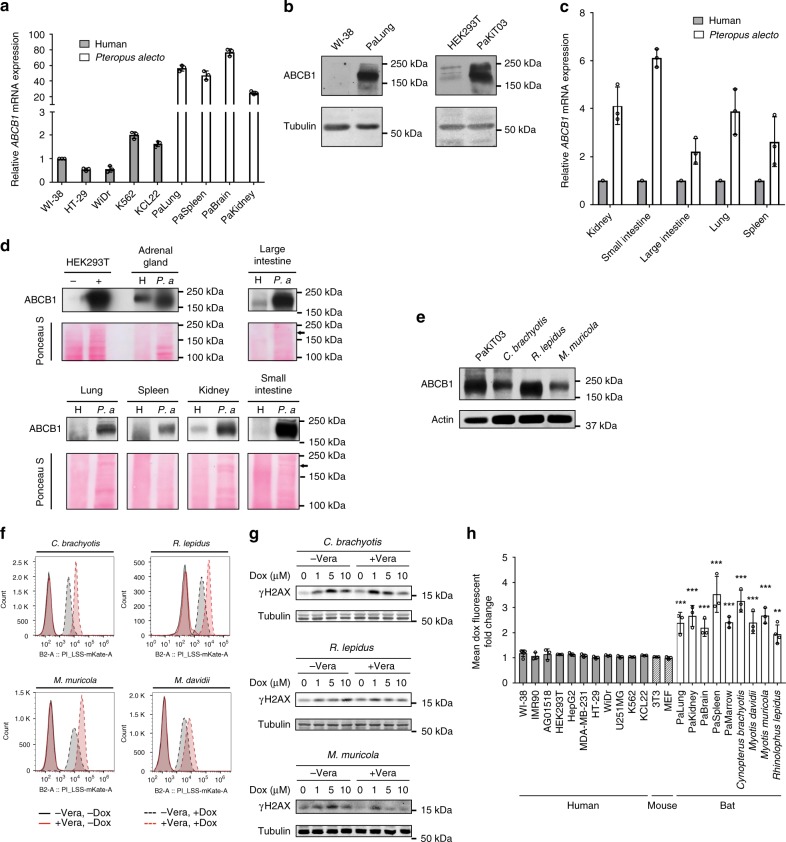
ABCB1 expression and efflux capability in human and multiple bat species. **a** qRT-PCR for *ABCB1* mRNA expression in cell lines derived from human and *P. alecto*. *ABCB1* mRNA expression is normalised to *GAPDH* and presented relative to the expression in WI-38 cells (mean ± SDs of three independent experiments). **b** Western blot analysis of ABCB1 in the indicated cell lines. Tubulin was used as a loading control. **c** qRT-PCR for *ABCB1* mRNA expression in tissues derived from human (*N* = 1) and *P. alecto* (*N* = 3). *ABCB1* mRNA expression is normalised to *GAPDH* and presented relative to the respective human tissues (mean ± SDs). **d** Western blot analysis of ABCB1 protein in the indicated tissues. Ponceau S staining of the membrane is shown as a loading control. Arrow indicates the size of ABCB1 protein. H and P. a indicate human and *P. alecto* respectively. Lysates from HEK293T transfected with control (−) or ABCB1 expressing plasmid (+) serve as negative and positive control, respectively. **e** Western blot analysis of ABCB1 in cells derived from the different bat species. Actin was used as a loading control. **f** Flow cytometry analysis of doxorubicin accumulation in cells derived from different bat species. Cells were treated and analysed as in Fig. 3b. **g** Western blot analyses of γH2AX in cells derived from different bat species. Cells were treated as in Fig. 4. Blots are representative of three independent experiments. **h** Flow cytometry analysis of doxorubicin accumulation. Cells were pre-treated without or with 5 μM verapamil (Vera) for 30 min before the treatment with 10 μM doxorubicin alone or together with Vera for an additional 3 h. The fold change by Vera treatment is presented as the mean fluorescence intensity of cells treated with both verapamil and doxorubicin relative to the mean fluorescence intensity of cells treated with doxorubicin alone. Bars represent mean fold change ± SD of at least three experiments. Data shown in Figs. 3b, c and 7f are also included in this histogram. Statistical significances were calculated using unpaired student’s two-sided *t* test. **p < 0.01, ***p < 0.001

To determine if *ABCB1* is differentially expressed in *P. alecto* compared to human cells, we first performed RT-qPCR on a panel of cell lines. Consistent with our RNA-seq results from PaKiT03 and HEK293T cells (Fig. 5a), *ABCB1* transcript levels were significantly higher in various *P. alecto* cell lines than in various human cell lines (Fig. 7a). Further, ABCB1 protein levels were higher in PaLung and PaKiT03 cells compared to human WI-38 and HEK293T cells (Fig. 7b). Next, we examined ABCB1 transcript and protein levels in various normal tissues from *P. alecto* and human. We found that ABCB1 levels are higher in *P. alecto* than in human across multiple tissues (Fig. 7c, d), including tissues known to express ABCB1 in human (adrenal gland, kidney, small intestine and large intestine) as well as those that express no or very little ABCB1 in human (lung and spleen). These data suggest that ABCB1 is expressed at higher levels and more broadly in *P. alecto* compared to human.

A comparison of *P. alecto* ABCB1 amino acid sequence with the human sequence demonstrated similarity of 89.5% (Supplementary Table 1). In addition, Walker A-, Walker B-, and active transport signature-motifs, which are characteristic of ABC transporters, were 100% conserved across multiple phylogenetically distant mammals (Supplementary Fig. 4), indicating that the ABCB1 amino acid sequence is well-conserved among mammals. Therefore, the increased drug efflux via ABCB1 in *P. alecto* cells compared to human cells is likely due to the higher expression of ABCB1 rather than the amino acid sequence differences.

### ABCB1-mediated efflux capability is conserved in multiple bat species

To determine if other families of bats also possess ABCB1-dependent drug efflux, we investigated cells derived from *Cynopterus brachyotis* (*Pteropodidae* family), *Rhinolophus lepidus* (*Rhinolophidae* family) and *Myotis muricola* and *Myotis davidii* (*Vespertilionidae* family), which have distinct maximum lifespans and body masses (Supplementary Table 2). Cells from these bat species showed high expression of ABCB1 protein, similar to *P. alecto* (*Pteropodidae* family) (Fig. 7e), as well as efficient doxorubicin efflux that depended on ABC transporter activity (Fig. 7f). Further, these cells displayed the induction of γH2AX by doxorubicin, but pre-treatment with verapamil led to a doxorubicin dose-dependent decrease in γH2AX levels (Fig. 7g), consistent with elevated levels of intracellular doxorubicin and histone eviction upon inhibition of ABCB1.

To further confirm that ABCB1-mediated drug efflux is unique to bats, we extended our analysis to a diverse range of cell lines from bats, mice, and humans. We examined doxorubicin accumulation in the presence or absence of verapamil by flow cytometry in 11 human cell lines, 2 mouse cell lines, 5 *P. alecto* cell lines, and 4 cell lines from other bat species (Supplementary Table 3). Verapamil pre-treatment increased doxorubicin accumulation in all of the bat cell lines, but not in any of the human and mouse cell lines (Fig. 7h). Together, these results suggest that high ABCB1 expression and enhanced drug efflux capacity are uniquely conserved in multiple bat species and may contribute to reducing DNA damage and perhaps, in turn, cancer incidence in bats.

## Discussion

Emerging evidence suggests that several long-lived mammals possess unusually low rates of cancer occurrence^2^. Technological advances in genomic sequencing have facilitated analysis of these organisms and revealed unique mechanisms of cancer resistance that are not observed in human^2^. Bats are long-lived mammals with an extremely low incidence of cancer^2,10^. However, the mechanisms underlying their cancer resistance are not fully understood. We have access to the bat species *P. alecto*, a large, non-hibernating fruit bat of the *Pteropodidae* family. Using cell lines established from *P. alecto*, we discovered that *P. alecto* cells uniquely displayed increased resistance to DNA damage induced by specific chemicals. This resistance is due at least in part to increased efflux of the chemicals mediated by high and broad expression of the ABCB1 transporter.

ABC transporter family proteins regulate cellular drug efflux^32^. ABCB1, also known as the transmembrane protein P-glycoprotein (P-gp) and multidrug resistance protein 1 (MDR1), is one of the best-characterised efflux transporters and is highly conserved across mammals^37^. In mammals, ABCB1 is specifically expressed in regions of detoxification, excretion, and protective barriers such as the intestinal epithelium, lumens of the liver, proximal tubule of the kidney as well as blood-tissue barriers of the brain. In rodents, the *ABCB1* gene encodes two isoforms, *Abcb1a* (*Mdr1a*) and *Abcb1b* (*Mdr1b*). Mice deleted for *Abcb1a* alone or both *Abcb1a* and *Abcb1b* are viable and not cancer-prone in laboratory conditions, which are free from genotoxic substances. However, they are more sensitive to toxins than wild-type mice^38,39^. We found that *P. alecto* expresses ABCB1 relatively highly and in additional tissues compared to human, such as lung and spleen. The broad expression of ABCB1 may contribute to more efficient protection from genotoxic substances in their environment.

ABCB1 was originally discovered in cancer cells where its high and functional expression promotes resistance to a variety of chemotherapeutic drugs, such as paclitaxel, vinblastine, etoposide and doxorubicin^32,34,40^. This broad chemoresistance is achieved by the promiscuous substrate specificity of ABCB1. Cancer cells acquire ABCB1 overexpression after prolonged chemotherapy, which is one of the major causes of cancer relapse since ABCB1-mediated drug efflux can reduce drug intake^32,34^. Likewise, generating cancer cell lines with high ABCB1 expression to study the biological implications of ABCB1 often requires prolonged exposure of cells to drugs for months^41,42^. It is, therefore, rather intriguing that various bat cell lines from many different tissues intrinsically possess and maintain high-ABCB1 expression and drug efflux in the absence of known selective pressure. Notably, in addition to xenobiotics, ABCB1 is reported to transport endogenous compounds such as cytokines, short peptide and lipids^43,44^. It is possible that bat cells may possess endogenous substances that are ABCB1 efflux substrates, and thus express high levels of ABCB1 to maintain homoeostasis.

ABCB1 expression is regulated by multiple signalling pathways and physiological factors. Previous studies have reported that c-Myc, c-Jun, HIF-1 and CtBP1 can positively modulate ABCB1 expression in cancers, leading to the development of drug resistance^45^. Recent work on microRNA biology (miRNA) such as miR-27a^46,47^, miR-223^48^ and miR-451^46^, have also illustrated their potential roles in regulating ABCB1 expression. Furthermore, physiological substances including steroid hormones, environmental stresses such as temperature, osmotic pressure and pH, and xenobiotics such as insecticides, heavy metal and DNA damage reagents have been shown to significantly modulate ABCB1 expression *in vitro*^45^. Interestingly, human pathogenic virus and virus proteins are also reported to induce ABCB1 expression^49,50^. Since bats are renowned for being a reservoir of high mortality viruses like Ebola^51^ and SARS^52^, some viral proteins may induce ABCB1 expression. It remains to be elucidated how bat cells induce and retain the high expression of ABCB1.

High doses of γ-irradiation, such as 10 Gy used in this study, mainly cause DNA DSB^53^. DSBs are the most detrimental form of DNA breaks for cells, and most DSBs are repaired via non-homologous end-joining (NHEJ)^26,54^. γH2AX and 53BP1 protein are core components of the NHEJ pathway. Once phosphorylation of H2AX occurs in response to DSBs, 53BP1 rapidly forms foci and disappears upon repair. We observed that 53BP1 foci formation and clearance after 10 Gy of γ-irradiation were fairly comparable between the cells from *P. alecto* and human, whereas mouse cell displayed a slower 53BP1 response. On the other hand, γH2AX signals induced by the same treatment were similar between bat, human and mouse cells. Why there is a delay between γH2AX phosphorylation and 53BP1 foci formation in mouse cells compared to human and bat cells remains to be deciphered.

Nucleotide excision repair (NER) and base excision repair (BER) are utilised to repair single-strand breaks such as removing bulky DNA adducts caused by UV irradiation^55^. Podlutsky et al.^56^ reported that human and bat cells have better BER than mouse cells, whereas human cells have better NER than bat and mouse cells. Our data suggest that human and bat cells have a better response to DSBs compared to mouse cells. It is likely that each species has evolved different strategies to cope with the different types of DNA damage.

Doxorubicin was originally isolated from a soil bacterium and can induce DSBs and single-stranded breaks via multiple mechanisms, including DNA intercalating to inhibit topoisomerase II activity, histone eviction, and induction of oxidative stress^30,57^. The mechanisms of cell death induced by doxorubicin are cell context-dependent and could be mediated by DNA damage or be independent of DNA damage such as replication stress or oxidative stress to proteins and other organelles^57^. Importantly, our data show that bat cells accumulate less doxorubicin compared to human and mouse cells, leading to reduced DSBs, and that ABCB1 is responsible for the reduced doxorubicin accumulation in bat cells.

Etoposide was originally isolated from plants and forms a ternary complex with DNA and topoisomerase II, thereby causing DNA DSBs^29^. In our experiments, we found that γH2AX resolved faster in *P. alecto* cells than in human and mouse cells when transiently treated with etoposide. Etoposide is a well-known ABCB1 substrate, therefore human and mouse cells would be expected to have higher residual intracellular etoposide than *P. alecto* cells even after drug removal from the media, leading to more DNA damage and γH2AX accumulation than *P. alecto* cells. Thus, the prolonged γH2AX in human and mouse cells could reflect not only DNA repair but also DNA damage caused by residual etoposide. Similar to doxorubicin, the assessment of DNA repair caused by etoposide between the *P. alecto*, human and mouse cells is challenging since the amounts of intracellular etoposide are not equal among these animals.

Other mechanisms of cancer resistance in bats have been proposed previously. For example, genome studies of the longest-lived bat *Myotis brandtii* showed that cancer resistance might be attributed to growth suppression by specific mutations in the growth hormone receptor that may reduce the GH-insulin-like growth factor 1 signalling pathway^58^. In addition, miRNA analysis revealed that three tumour suppressive miRNAs (miR-101-3p, miR-16-5p and miR-143-3p) were upregulated and a single tumour promoting miRNA (miR-221-5p) was downregulated in *Myotis myotis*^59^. These data suggest that bats may have multiple strategies to prevent tumour formation.

Unlike conventional mouse models, in vivo validation of our proposed mechanism in bats is hindered by their low reproductive capacity and technological limitations for genetic manipulation^10,60^. Since the function of ABCB1 in mice was reported to be limited to specific tissues^38,39^, it would be of interest to determine whether transgenic mice with high and broad expression of *ABCB1* are less tumour prone, particularly in response to ABCB1 substrates.

Laboratory-bred animals and their wild-caught counterparts likely respond differently to xenobiotic and environmental challenges^61–63^. *ABCB1* knockout mice are not cancer-prone in the laboratory conditions where is free from genotoxic substances but are more sensitive to toxins than wild-type mice^39^. Therefore, whether *ABCB1* knockout mice are cancer-prone in the wild remains to be investigated. Harper et al. have shown that cells derived from wild-trapped mice exhibited different sensitivity to various genotoxic insults than cells derived from laboratory-bred mice^62^. Hence, we do not exclude the possibility that the environmental background could contribute to the differences we observed between bats and mice. It would be interesting to compare the ABCB1-mediated efflux capacity of bat cells to cells derived from wild mice.

In summary, using *P. alecto* as a model, we demonstrated that bats have a high and broad expression of ABCB1, which uniquely promotes resistance to DNA damage induced by specific chemicals. This phenotype is conserved in multiple bat species, possibly as a pan-bat biological feature, and might partly explain their low incidence of cancer. Future research into this potential tumour suppressor mechanism in bats could inform innovative strategies to prevent or treat human cancer, as well as chemoresistance.

## Methods

### Cell culture

*P. alecto* and *M. davidii*-derived cell lines listed in Supplementary Table 3 and were established as previously described^18,64^. Briefly, the tissues were first cut finely using a scalpel, washed with cold media and further processed by enzymatic reactions for primary cell culture. *C. brachyotis*, *M. muricola* and *R. lepidus* were captured in Singapore under the National Parks Board permit NP/RP11-011 and NP/RP14-109. Lung tissues were harvested to produce primary cell cultures. Lung tissues were sliced into 1 mm sized pieces and treated enzymatically (1X HBSS, 0.2% bovine serum albumin, 0.4 mg/mL collagenase-A, 0.22 µm filtered) and incubated at 37 °C with rotation (60 rpm) for approximately 1 h to dissociate cells. Dissociated tissues were centrifuged at 430×*g* for 5 min, resuspended with complete RPMI medium 1640 (10% foetal bovine serum (Thermo Scientific), 2% penicillin/streptomycin (Thermo Scientific), 2.5% HEPES) and plated in T-25 tissue culture flasks. Primary lung cells were maintained at 37 °C with 5% CO_2_ until confluence. After the second passaging, all cell lines were grown in Dulbecco’s modified Eagle’s medium (DMEM) with 10% foetal bovine serum and penicillin/streptomycin in a 37 °C incubator with 5% CO_2_, with exception of K562 and KCL22 cells that were maintained in RPMI medium 1640 with 10% foetal bovine serum and penicillin/streptomycin. Primary cell lines from the tissues of other bat species were established using identical method for lung tissues. Origin of cell lines and immortalisation type are listed in Supplementary Table 3. Tissue samples of the adrenal gland, lung, spleen, kidney, small and large intestines were obtained from *P. alecto* and were stored in Buffer RLT (Qiagen) at −80 °C for protein extraction. WI-38, IMR-90 and AG01518 cell lines were purchased from Coriell Institute. HEK293T, HepG2, MDA-MB-231, HT-29, WiDr, U251MG, K562 and NIH3T3 cell lines were purchased from American Type Culture Collection (ATCC). KCL-22 cell line was purchased from Leibniz Institute DSMZ-German. MEF cells were kindly provided by Dr Yanping Zhang (University of North Carolina at Chapel Hill).

### Reagents

Doxorubicin (D4035), etoposide (E1383), cisplatin (P4394), verapamil hydrochloride (V4629) and cyclosporin A (C3662) were obtained from Sigma-Aldrich.

### Western blotting and antibodies

Protein extraction from cultured cells was conducted in sodium dodecyl sulfate (SDS) lysis buffer (50 mM Tris–HCl pH 6.8, 2% SDS, 10% glycerol). Protein extraction from *P. alecto* tissues was performed in RIPA lysis buffer (10 mM Tris-HCl pH 7.6, 150 mM NaCl, 1% TritonX-100, 1 mM EDTA) supplemented with EDTA-free complete protease inhibitor cocktail (04693132001, Roche), phosphatase inhibitor cocktail 2 (P5726, Sigma-Aldrich), and phosphatase inhibitor cocktail 3 (P0044, Sigma-Aldrich) using cell pellet homogeniser (VWR) on ice. Protein lysates extracted from the human adrenal gland (sc-363761), lung (sc-363767), spleen (sc-363779), kidney (sc-363764), small intestine (sc-364225) and large intestine (sc-363757) were purchased from Santa Cruz biotechnology, Inc. Lysates were then quantified, and equal amounts of protein were loaded for Western blotting analyses. Antibodies used are as follow: γH2AX (05-636, Millipore, 1:3000), tubulin (ab44928, Abcam, 1:5000), actin (MAB1501, Millipore, 1:10,000), ABCB1 (sc-8313 H241, Santa Cruz, 1:1000), horseradish peroxidase (HRP)-conjugated secondary antibody (Thermo Scientific, 1:10,000), and fluorescence labelled secondary antibodies (Thermo Scientific, 1:10,000). Detection of γH2AX, tubulin and actin were performed using LI-COR Odyssey (LI-COR Biosciences) or HRP substrate (Thermo Scientific). ABCB1 was detected using HRP substrate (Thermo Scientific). Uncropped western blotting images are provided in the Supplementary Information.

### Measurement of 53BP1 foci formation

Detection and quantification of 53BP1 foci formation were conducted using immunofluorescence and fluorescence microscopy. For immunofluorescence, PaLung, WI-38 and MEF cells were treated with either 10 Gy of γ-irradiation or 5 µM doxorubicin for 3 h before subjected for analysis at the respective time points. In brief, cells were fixed with 4% formaldehyde (Sigma-Aldrich) and permeablized with 0.2% Triton X-100 (Bio-Rad) followed by staining with anti-53BP1 antibody (sc-22760, Santa Cruz, 1:250), Alexa Fluor 488-conjugated anti-rabbit IgG (Jackson ImmunoResearch, 1:100), and DAPI (Sigma-Aldrich). Imaging of cells was performed using Olympus IX83 fluorescent microscope at 405 nm for DAPI and 488 nm for 53BP1. As for quantification of 53BP1 foci, analysis of cells was carried out using cell image analysis software CelProfiler^TM^ with the speckle counting pipeline^65^. A minimum of 100 cells was quantified per time point for each treatment condition. Average number of 53BP1 foci per cell was calculated using the equation: average number of 53BP1 foci per cell = (total number of foci)/(total number of cells quantified).

### Measurement of intracellular doxorubicin accumulation

Accumulation of intracellular doxorubicin was monitored or measured by intrinsic fluorescence of doxorubicin using either fluorescence microscopy or flow cytometry. For fluorescence microscopy, PaLung, PaKiT03, WI-38, HEK293T and MEF cells were pre-treated with or without 5 µM verapamil for 30 min, followed by incubation with doxorubicin for 3 h. Detection of doxorubicin in live cells was performed using Olympus IX71S1F3 fluorescent microscope. As for flow cytometry analysis, cells were pre-treated with or without 5 µM verapamil or cyclosporin A for 30 min, followed by incubation with doxorubicin for 3 h. Cells were then trypsinized, washed in PBS once and resuspended in PBS for analysis by MACSQuant Analyser 10 (Miltenyi Biotec). Laser excitation was set at 488 nm and filter with wavelengths 614/50 nm was used for detection. A total number of 30,000 live cells were analysed per sample. For Fig. 2e, f, and Supplementary Fig. 2D and E, the calculation was done using the equation: normalised doxorubicin fluorescence value = (mean fluorescence value)/(average cell volume). For Fig. 7h, the calculation was done using the equation: mean doxorubicin fluorescence fold change = (mean fluorescence with efflux inhibition)/(mean fluorescence without efflux inhibition).

### Cell volume determination

Mean volume (µL) of WI-38, PaLung, MEF, HEK293T and PaKiT03 cells were determined by Millipore Sceptre cell counter. Trypsinized cells were washed with PBS, before being measured using 60 µm sceptre tips (Millipore).

### Comet assay (DNA damage measurements in cells)

DNA damage in PaKiT03 cells was assessed by neutral comet assay as described^66^. Comets were stained with propidium iodide for 30 min before subjected for analysis. Images were acquired with a 20× objective lens. A minimum of 50 comets was assessed per treatment condition. Analysis of the comets and DNA damage calculated by olive tail moments were performed using CASPlab software^67^. Olive tail moments were calculated using the formula: olive tail moments = (the percentage of DNA in comet tail) × (distance between comet head and tail centres of gravity).

### Cell viability assay

PaKiT03 cells were seeded at 10,000 cells per well in a 96-well plate and kept overnight to allow attachment. Cells were pre-treated with or without 5 µM verapamil for 30 min, followed by co-treatment of increasing dose of doxorubicin (0.5, 1, 2.5, 5 and 10 µM doxorubicin) and verapamil for 3 h. Doxorubicin was then removed, and cells were replaced in doxorubicin-free medium with or without verapamil for 72 h before measuring cell viability using CellTiter-Glo^®^ luminescent cell viability assay purchased from Promega.

### siRNA knockdown

Transient transfection of siRNAs was performed using ScreenFect A (Wako) according to manufacturer’s instructions. Concentrations of siRNA for control, *ABCB1*, *ABCC1* and *ABCG2* were set at 20 nM. Control siRNA was purchased from Integrated DNA Technologies (NC1). Sense strand sequence of the siRNAs for *ABCB1*, *ABCC1* and *ABCG2* are as follows:

*ABCB1* #1 (5′-GAGCUUGAAAGGUACAACAAAAAdTdT-3′),

#2 (5′-GAUGAAGCUACAUCAGCUCUAGAdTdA-3′);

*ABCC1* #1 (5′-CAAACAGCAUCACCGUGAAAAACdGdC-3′),

#2 (5′-CUGGAAGAAGGAAUGUGCCAAAUdCdC-3′);

*ABCG2* #1 (5′-CAUGAAUAUAUCAGUGGAUACUAdCdA-3′),

#2 (5′-GGAGAAGAAUUCUUGAUAAAGCAdGdG-3′).

### Quantitative RT-PCR analysis

Total RNA from cell lines and tissue samples was extracted using TRIzol (Life Technologies) and RNeasy Mini Kit (Qiagen). A single panel of human tissue total RNA was purchased from Clontech (human total RNA Master Panel II #636643) and bat tissue total RNA was harvested from three individual *P. alecto*. All cDNA was synthesised using iScript cDNA synthesis kit (BioRad). Quantitative RT-PCR was performed with SYBR Green (KAPA Biosystems) using the CFX96 System (BioRad). Relative expression of each gene was calculated using *GAPDH* as an internal control by BioRad CFX manager software. The primers used are as follows:

*GAPDH* forward (5′-CGTATTGGGCGCCTGGTCAC-3′) and

reverse (5′-CATGTAGTTGAGGTCAATGAAGGGGTC-3′);

*ABCB1* forward (5′-TGGTGTGGTGAGTCAGGAACCTG-3′) and

reverse (5′-ACAGCTTTCTCAATCTCATCCATGGTG-3′);

*ABCC1* forward (5′-CCCATGAGGAGCTGGATCCGG-3′) and

reverse (5′-CTTCCGGAATGGAGAAGGTGATGC-3′);

*ABCG2* forward (5′-GTGTGTCTTCTGTGGAGCTCTTTGTG-3′) and

reverse (5′-CCCACCTCTGCTTTCAATCCTACC-3′). QPCR products were verified by sequencing.

### RNAseq data analysis

Whole transcriptome shotgun sequencing data of PaKiT03 and HEK293T cells from our previous study was obtained from the NCBI Sequence Read Archive under the accessions [SRX661189] and [SRX665117]^33^. RNA-Seq reads were mapped using Tophat (version 1.3.2) and Bowtie (version 0.12.7). Fragments per kilobase million (FPKM) values were calculated using Cufflinks (version 1.2.1). Differential expression was calculated using DESeq2. FPKM values were normalised per gene to obtain relative expression values.

### Protein sequence alignment and conservation comparison

Alignment of different mammalian ABCB1 protein sequences was generated with Bio Edit programme. Percentages of identity with other mammalian ABCB1 were analysed with reference to human ABCB1 protein sequence using Vector NTI Advance version 11.5. In this study, amino acid sequences for ABCB1 were obtained for *Bos taurus*—cow (XP_010802430.1), *Canis lupus familiaris* – dog (NP_001003215.2), *Felis catus*—cat (NP_001164535.1), *Heterocephalus glaber*—naked mole rat (XP_012934158.1), *Homo sapiens*—human (NP_000918.2), *Macaca mulatta*—Rhesus monkey (NP_001028059.1), *Mus musculus*—mouse ABCB1A (NP_ 035206.2) and mouse ABCB1B (NP_035205.1), *M. davidii*—David’s myotis (XP_006779228.1), *P. alecto*—black flying fox bat (XP_006925783) and *Sus scrofa*—pig (NP_001295175.1).

### Database for different species of bats

Maximum lifespan and body mass of *P. alecto*, *C. brachyotis* were acquired from AnAge database of Animal Ageing and Longevity [http://genomics.senescence.info/species/]. For *M. davidii*, *M. muricola* and *R. lepidus*, maximum lifespan and body mass were determined by averaging *Myotis* genus and *Rhinolophus* genus, respectively using available information derived from the same database.

### Statistical analysis

All statistical analyses in this study were conducted using the unpaired student’s two-sided *t* test.

### Reporting summary

Further information on research design is available in the Nature Research Reporting Summary linked to this article.

## Supplementary information

Supplementary Information

Peer Review

Reporting Summary

Source Data

## Data Availability

The RNA-seq of PaKiT03 and HEK293T cell lines are available in National Center for Biotechnology Information with the accession code SRP044809 [https://www.ncbi.nlm.nih.gov/sra/SRX661189[accn]] and [https://www.ncbi.nlm.nih.gov/sra/SRX665117[accn]]. Details of the bat species used in this study can be found in [http://genomics.senescence.info/species/]. The source data underlying Figs. 1b, 2c–f, 5a–c, 6c, 6e, 6f, 7a, 7c and 7h and Supplementary Figs. 2C–E are provided as a Source Data file. All data are available from authors upon request.
