# Supplementary material for: ABCB1 protects bat cells from DNA damage induced by genotoxic compounds
Source: Nat Commun. 2019 Jun 27;10:2820. doi: 10.1038/s41467-019-10495-4 (PMC6597548; doi:10.1038/s41467-019-10495-4)
Supplement: Supplementary file 1 — Supplementary Information [file 41467_2019_10495_MOESM1_ESM.pdf]

ABCB1 protects bat cells from DNA damage induced by genotoxic compounds

Koh et al.

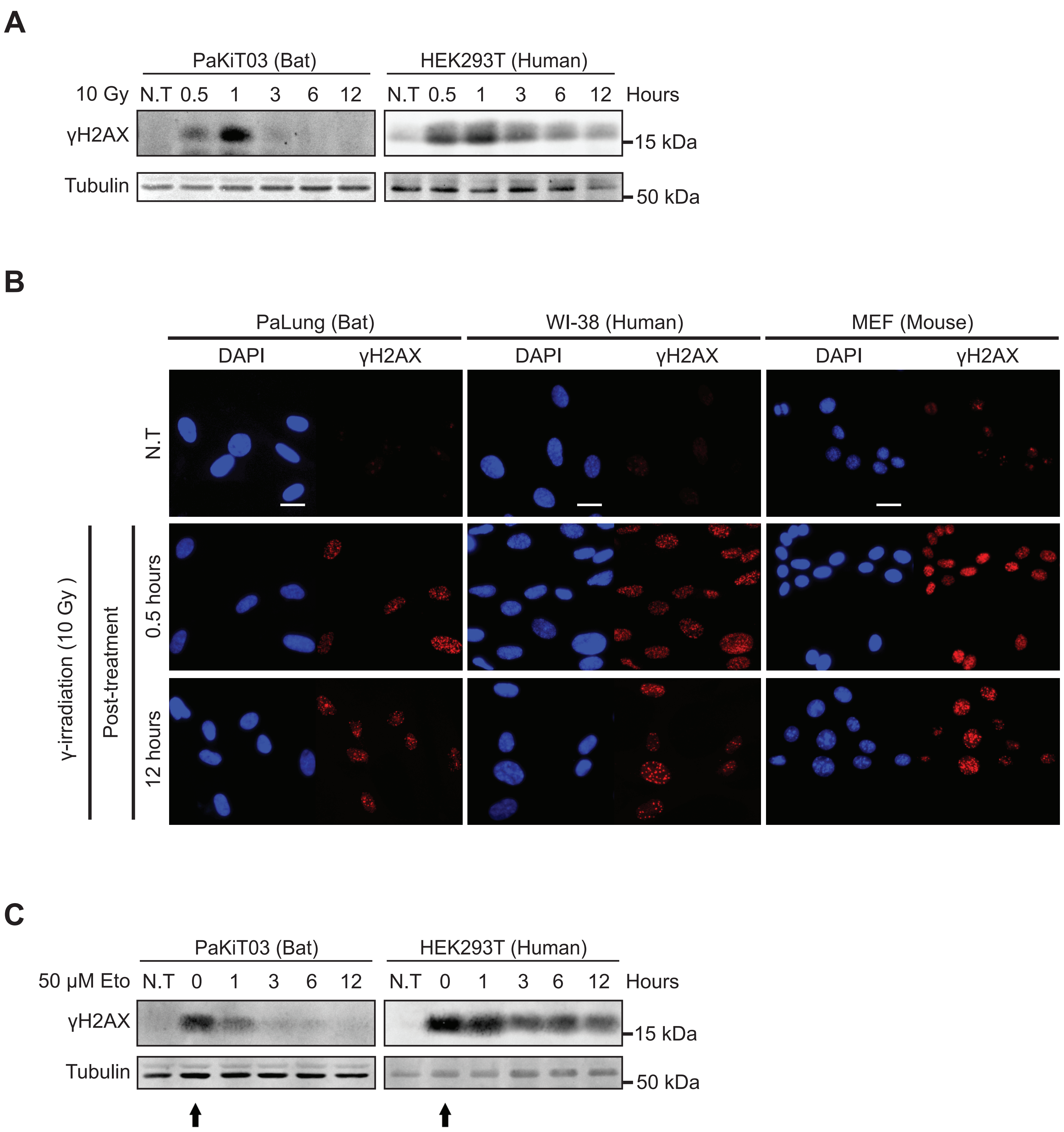

Supplementary Figure 1. Western blot analysis of γH2AX in *Pteropus alecto* and human cell lines treated with γ-irradiation

(A) Western blot analysis of γH2AX in PaKiT03 and HEK293T cells exposed to 10 Gy of γ-irradiation. Protein lysates were harvested at the indicated time points. Tubulin was used as a loading control. N.T stands for no treatment. Blots are representative of three independent experiments.

(B) Immunofluorescence staining of γH2AX for PaLung, WI-38 and MEF cells. Cells were treated with 10 Gy of γ-irradiation and fixed at the indicated time point. N.T stands for no treatment. Scale bars represent 20 µm. Results are representative of three independent experiments.

(C) Western blot analysis of γH2AX in PaKiT03 and HEK293T cells. Cells were treated with 50 µM etoposide (Eto) for 3 hours, followed by drug-free medium up to 12 hours (starting at t=0 hours, indicated by arrow). Protein lysates were harvested at the indicated time points. Tubulin was used as a loading control. N.T stands for no treatment. Blots are representative of three independent experiments.

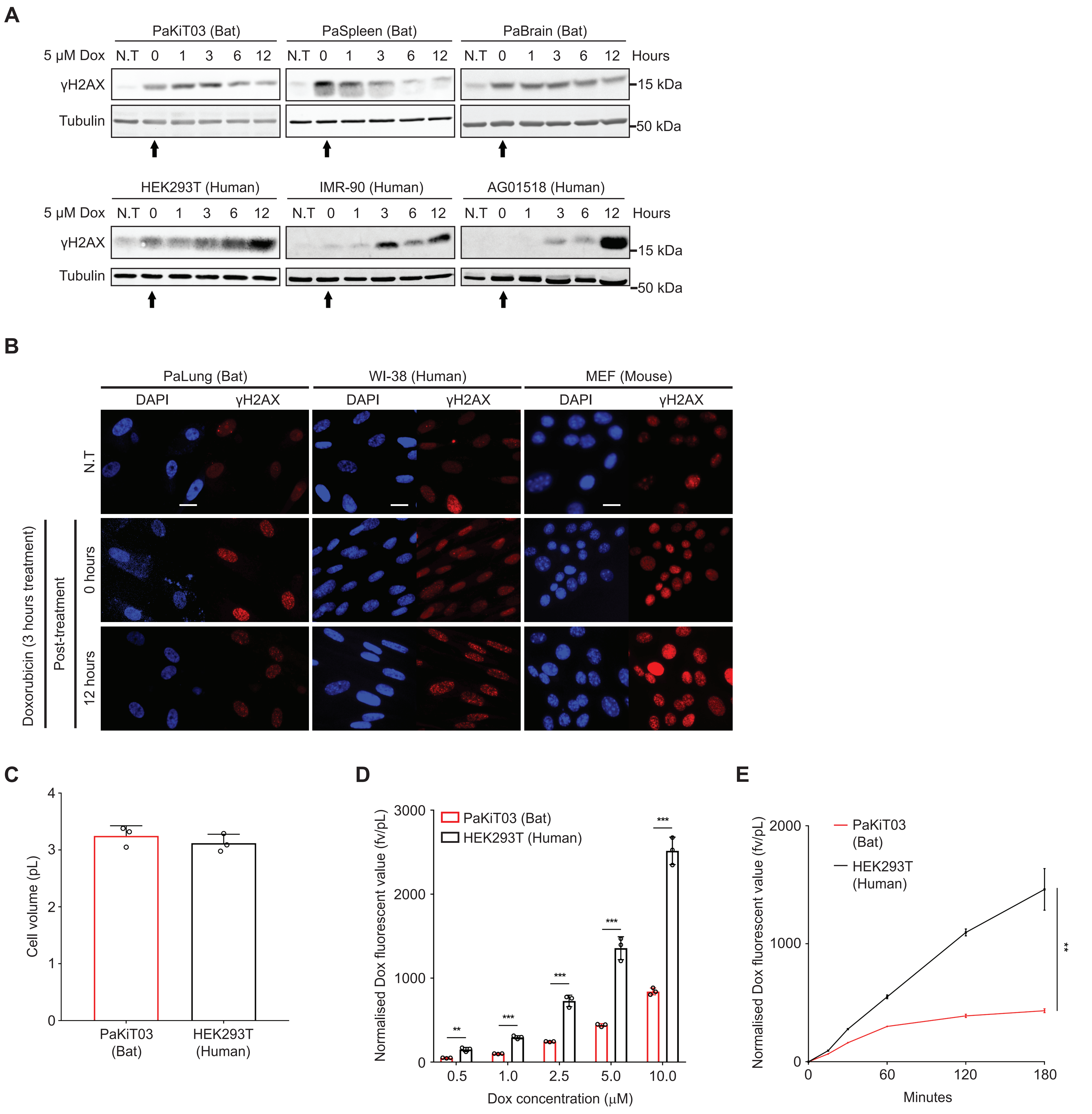

Supplementary Figure 2. Western blot analysis of γH2AX and intracellular accumulation of doxorubicin in *Pteropus alecto* and human cell lines

(A) Western blot analysis of γH2AX. The indicated cells were treated with 5 µM doxorubicin (Dox) for 3 hours, followed by drug-free medium up to 12 hours (starting at t=0 hours, indicated by arrow). Protein lysates were harvested at the indicated time points. Tubulin was used as a loading control. N.T stands for no treatment. Blots are representative of three independent experiments.

(B) Immunofluorescence staining of γH2AX for PaLung, WI-38 and MEF cells. Cells were treated with 5 µM doxorubicin for 3 hours, and fixed immediately (0 hour) or at 12 hours after culturing in a doxorubicin-free medium. N.T stands for no treatment. Scale bars represent 20 µm. Results are representative of three independent experiments.

(C) The volume of PaKiT03 and HEK293T cells. Bars represent mean volume ± SD of three experiments.

(D) Doxorubicin accumulation in PaKiT03 and HEK293T cells after 3 hours of incubation at the indicated concentration. The amount of accumulated doxorubicin in cells was analysed by flow cytometry. The mean fluorescence value (fv) of doxorubicin is normalised to cell volume (pL). Results were generated from three experimental repeats. Statistical significance of $p < 0.01$ was represented with ** and $p < 0.001$ with ***.

(E) Time course of doxorubicin accumulation in PaKiT03 and HEK293T cells. Cells were treated with 5 µM doxorubicin for the indicated time, and analysed by flow cytometry. The mean fluorescence value of doxorubicin accumulated in each cell line is normalised to cell size. Results were generated from three experimental repeats. Statistical significance of $p < 0.01$ was represented with **.

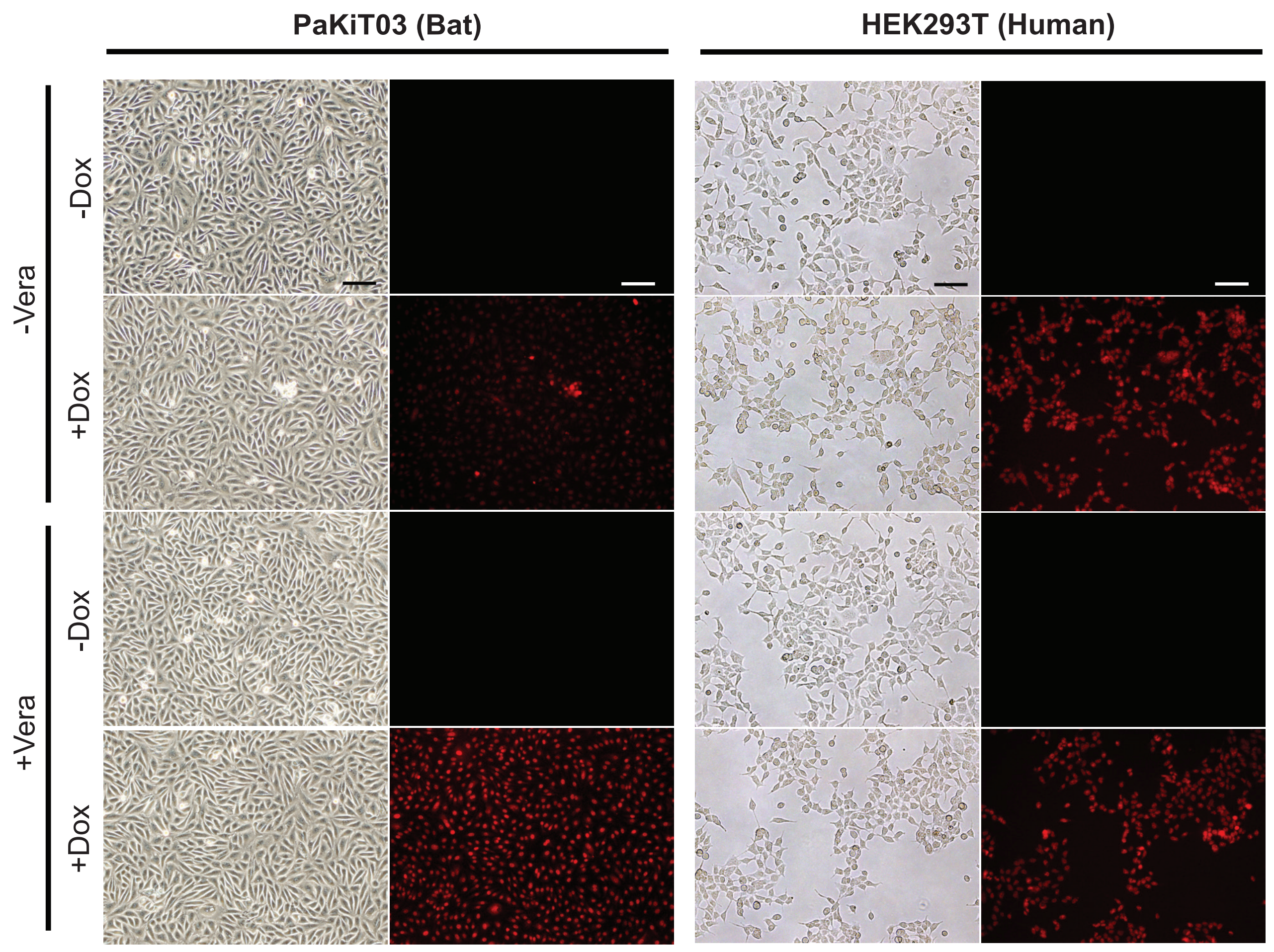

Supplementary Figure 3. Analysis of drug efflux capability via ABC transporters in PaKiT03 and HEK293T cells

Doxorubicin (Dox) fluorescence or phase contrast images of PaKiT03 and HEK293T cells. Cells were pre-treated without or with 5 μM verapamil (Vera) for 30 minutes before the treatment with 10 μM doxorubicin alone or together with verapamil for an additional 3 hours. Images were acquired with 10x objective lens and scale bar represents 100 μm. All results were representative of at least three experimental repeats.

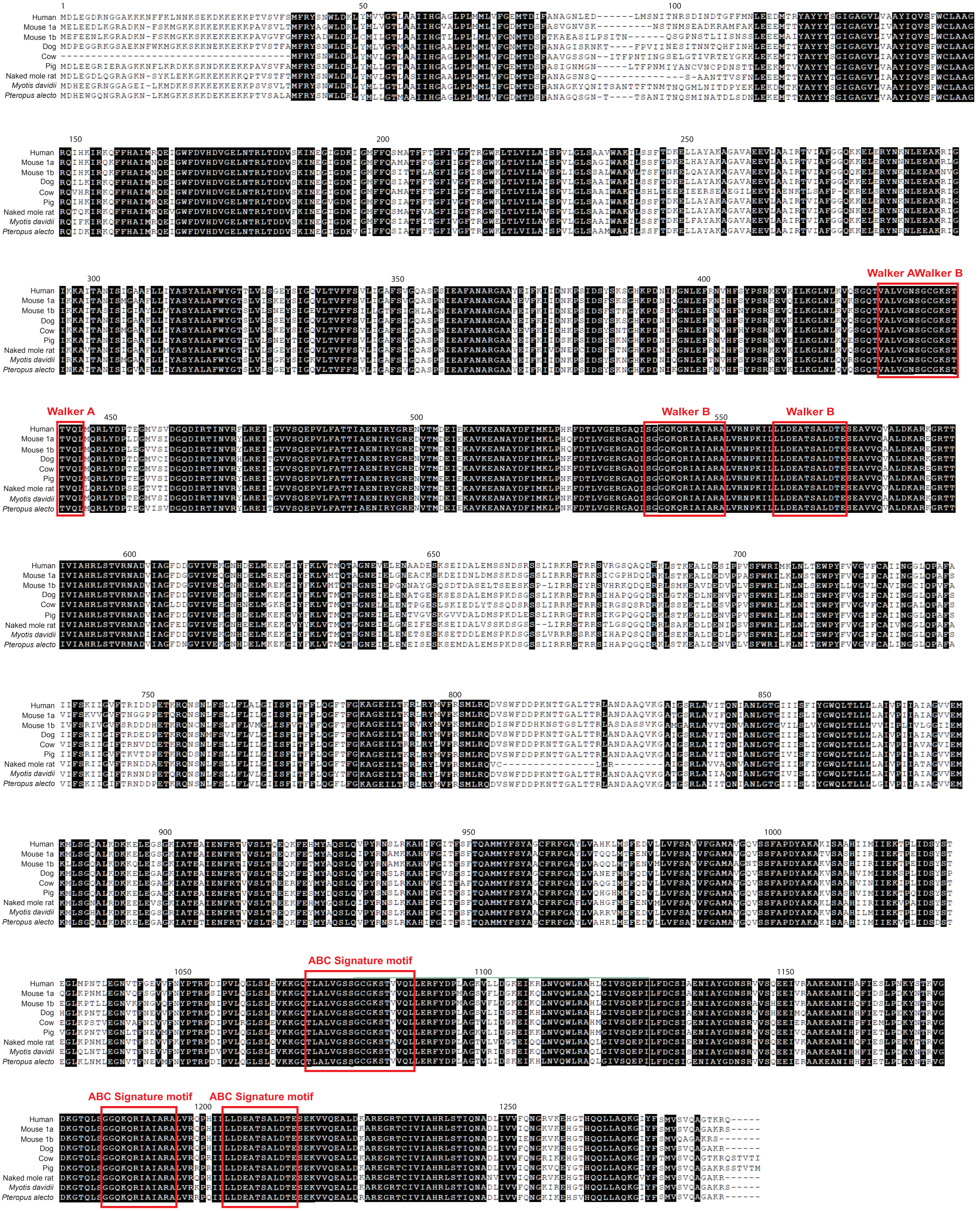

Supplementary Figure 4. A sequence alignment of ABCB1 protein

Amino acid sequences of ABCB1 from the indicated mammals are shown. nmr indicates naked mole rat. Amino acid residues conserved across species are highlighted in black. Walker A-, Walker B-, and ABC signature-motifs are marked with red rectangle.

Supplementary Table 1. Homology of ABCB1 protein sequence between human and other species

**Mammalian species**	**Percentage conserved to Human ABCB1 protein (%)**
Bat, *Pteropus alecto*	89.5
Bat, *Myotis davidii*	88.4
Rhesus monkey, *Macaca mulatta*	96.2
Mouse Abcb1a, *Mus musculus*	87.2
Mouse Abcb1b, *Mus musculus*	79.5
Dog, *Canis lupus familiaris*	90.0
Cow, *Bos taurus*	83.8
Pig, *Sus scrofa*	88.8
Cat, *Felis catus*	90.8
Naked mole rat, *Heterocephalus glaber*	84.8

Supplementary Table 2. Summary of individual or average maximum lifespan and body mass of bat species used in this study. All data are summarised from AnAge dataset, http://genomics.senescence.info/species/

**Bat species**	**Maximum lifespan (years)**	**Body mass (g)**
Pteropus alecto	19.7	672.1
Cynopterus brachyotis	10.1	45
Myotis species (Average)	22.13	10.4
Rhinolophus species (Average)	24.3	11.9

Supplementary Table 3. Summary of cell lines established and used in this study

Species	Cell lines	Description
Human	WI-38	Human fetal lung-derived fibroblast
	IMR-90	Human fetal lung-derived fibroblast
	AG01518	Normal human foreskin-derived fibroblast
	HEK293T	Human embryonic kidney epithelial cells, SV40 immortalized
	HepG2	Human hepatocellular carcinoma
	MDA-MB-231	Human adenocarcinoma
	HT-29	Human colorectal adenocarcinoma
	WiDr	Human colorectal adenocarcinoma
	U251MG	Human glioblastoma astrocytoma
	K562	Chronic myelogenous leukemia
	KCL22	Chronic myeloid leukemia in blast crisis
Mouse	NIH3T3	Mouse embryonic fibroblast, self-immortalized
	MEF	Mouse embryonic fibroblast, self-immortalized
Bats	PaLung	*Pteropus alecto* lung-derived fibroblast
	PaKidney	*Pteropus alecto* kidney-derived fibroblast
	PaBrain	*Pteropus alecto* brain-derived fibroblast, hTert immortalized
	PaSpleen	*Pteropus alecto* spleen-derived fibroblast, SV40 immortalized
	PaMarrow	*Pteropus alecto* bone marrow-derived cells, self-immortalized
	PaKiT03	*Pteropus alecto* kidney-derived fibroblast, SV40 immortalized
	C. brachyotis Lung	*Cynoterus brachyotis* primary lung-derived fibroblast, *Pteropodidae* family
	M. davidii Kidney	*Myotis davidii* kidney-derived cell line, self-immortalized, *Vespertilionidae* family
	M. muricola Lung	*Myotis muricola* Lung-derived cell line, self-immortalized, *Vespertilionidae* family
	R. lepidus Large Intestine	*Rhinolopus lepidus* primary large intestine-derived fibroblast, *Rhinolophidae* family

Figure 1A

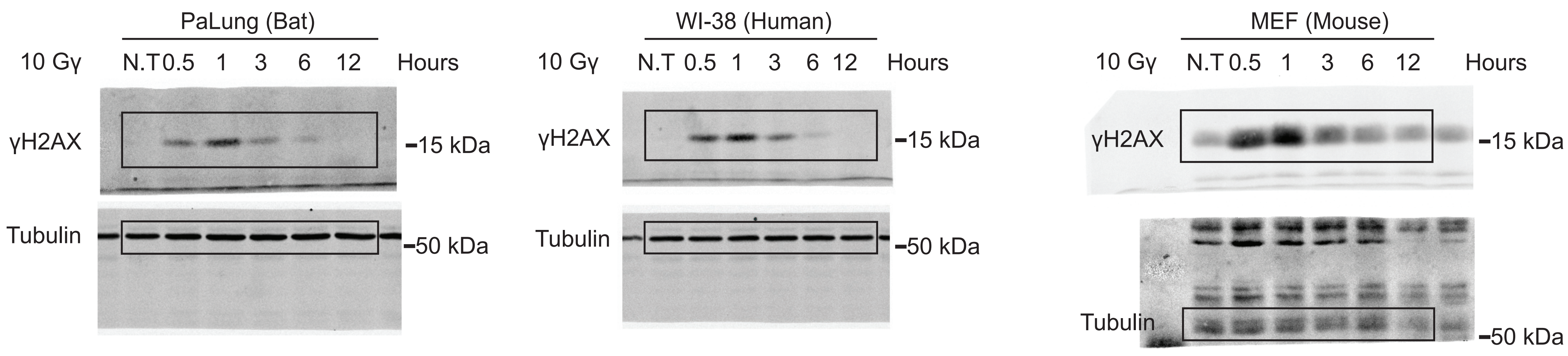

Figure 1C

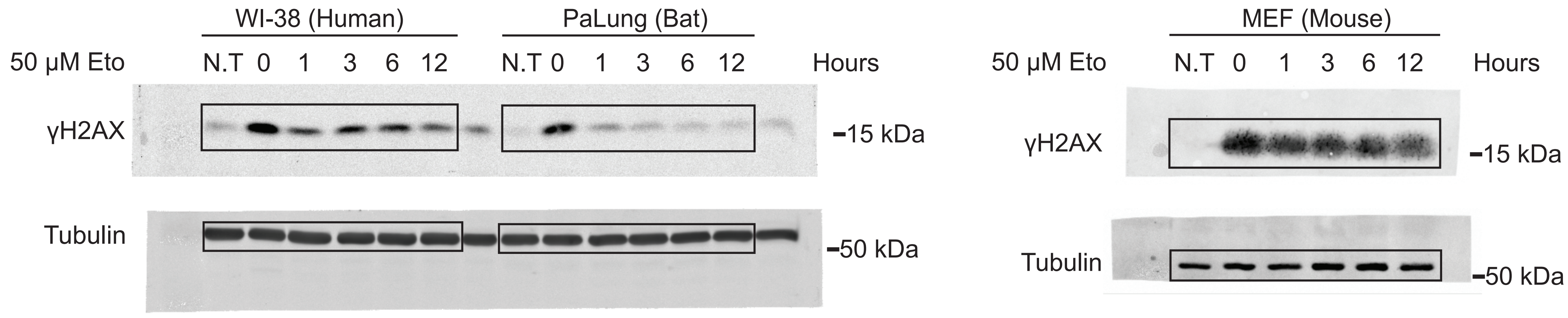

Supplementary Figure 5. Uncropped Western blots for Figure 1A and 1C

Figure 2A

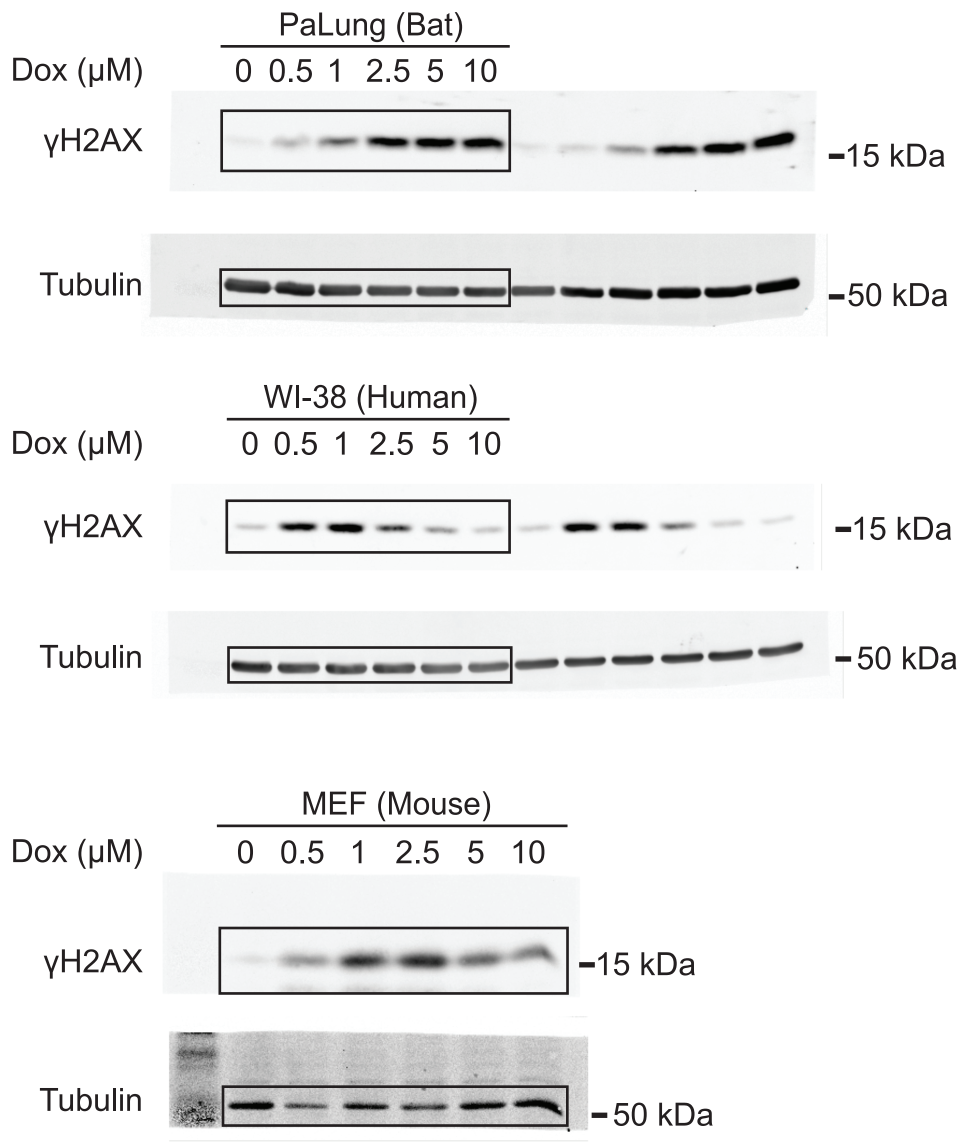

Figure 2B

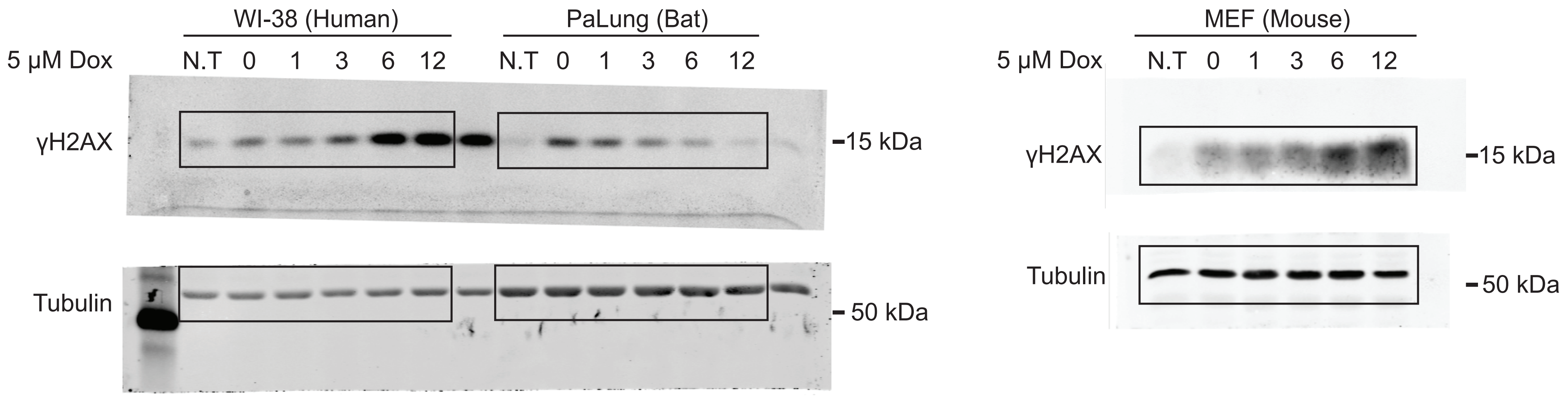

Supplementary Figure 6. Uncropped Western blots for Figure 2A and 2B.

Figure 4A

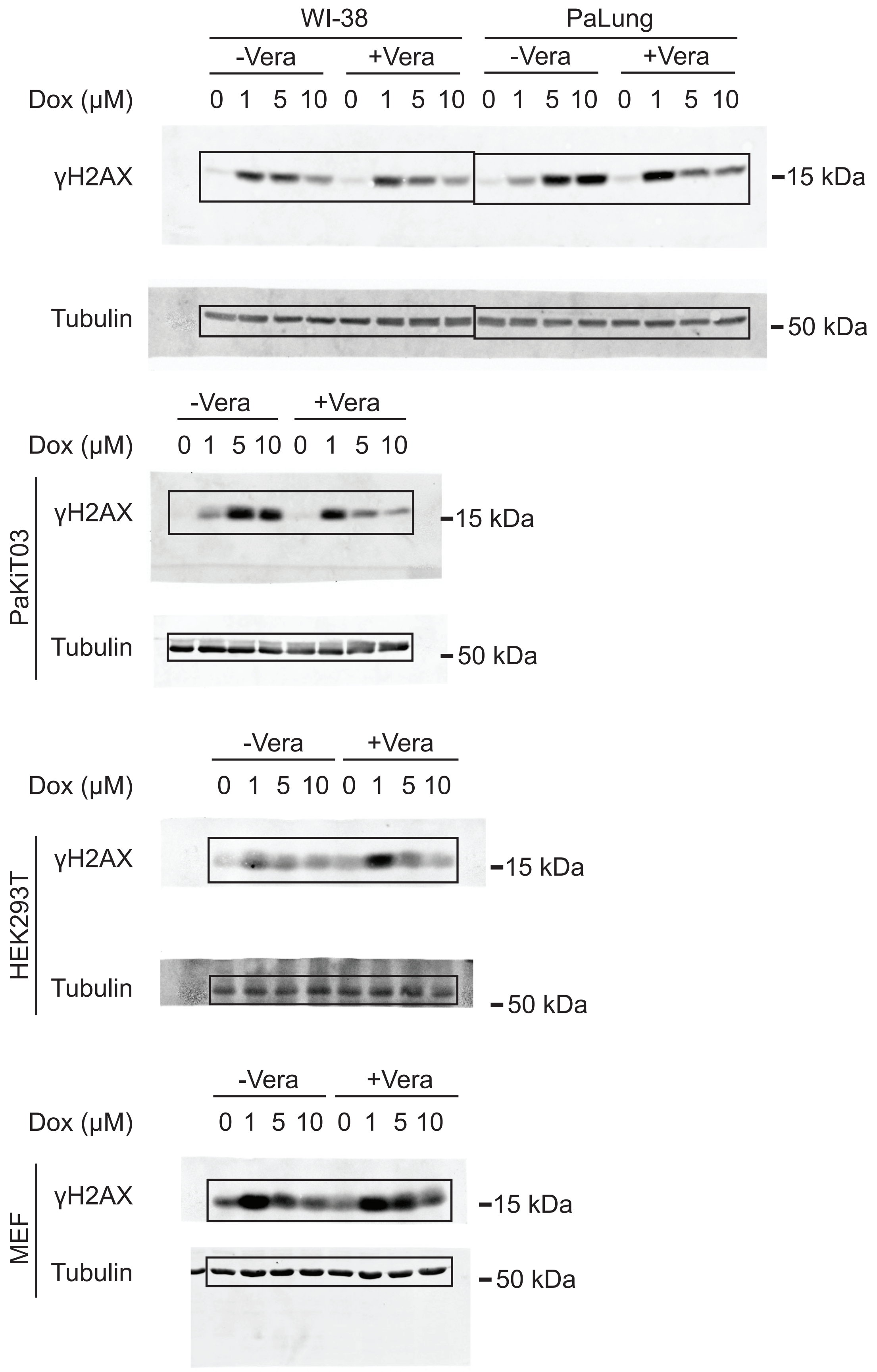

Figure 4B

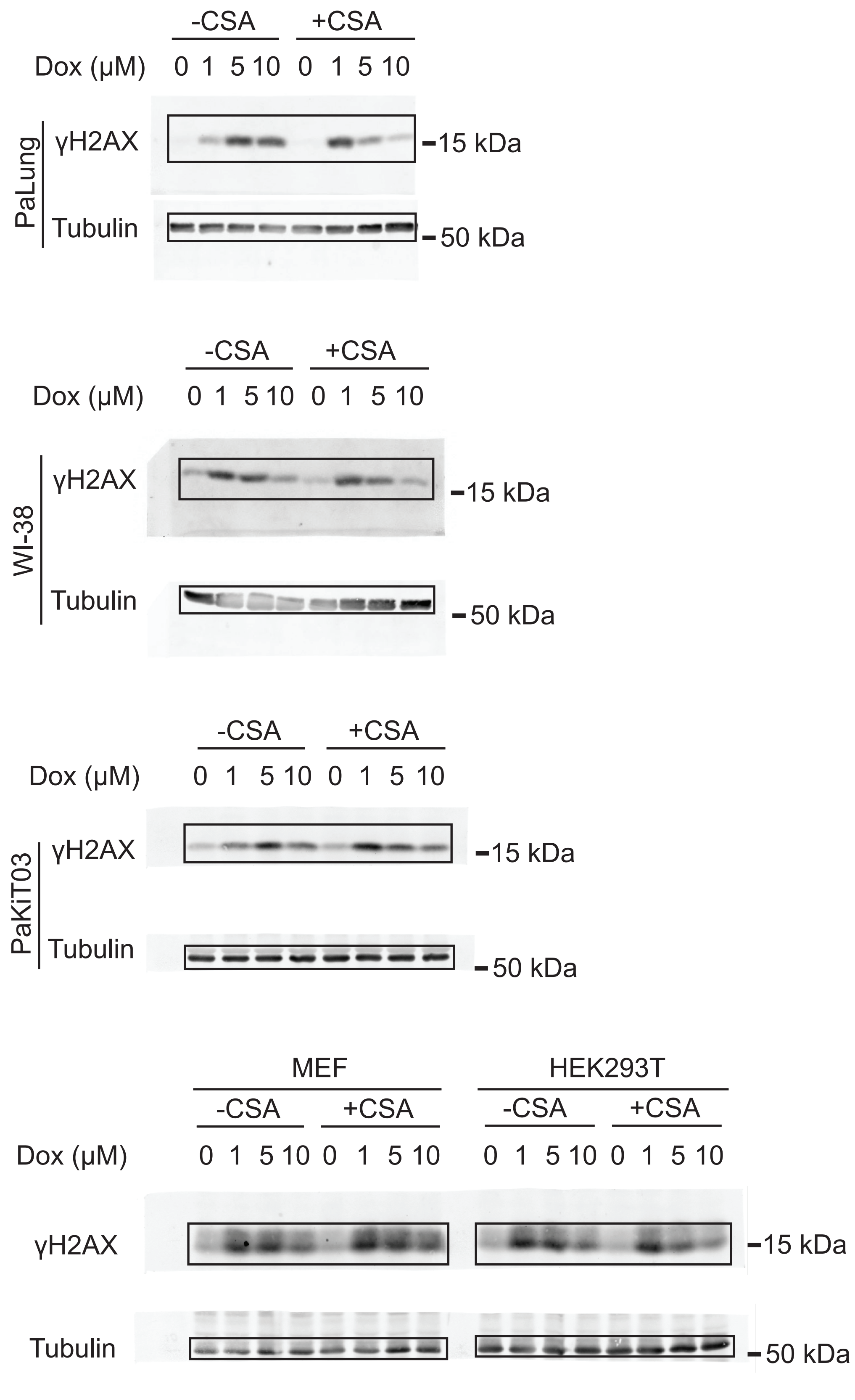

Figure 4C

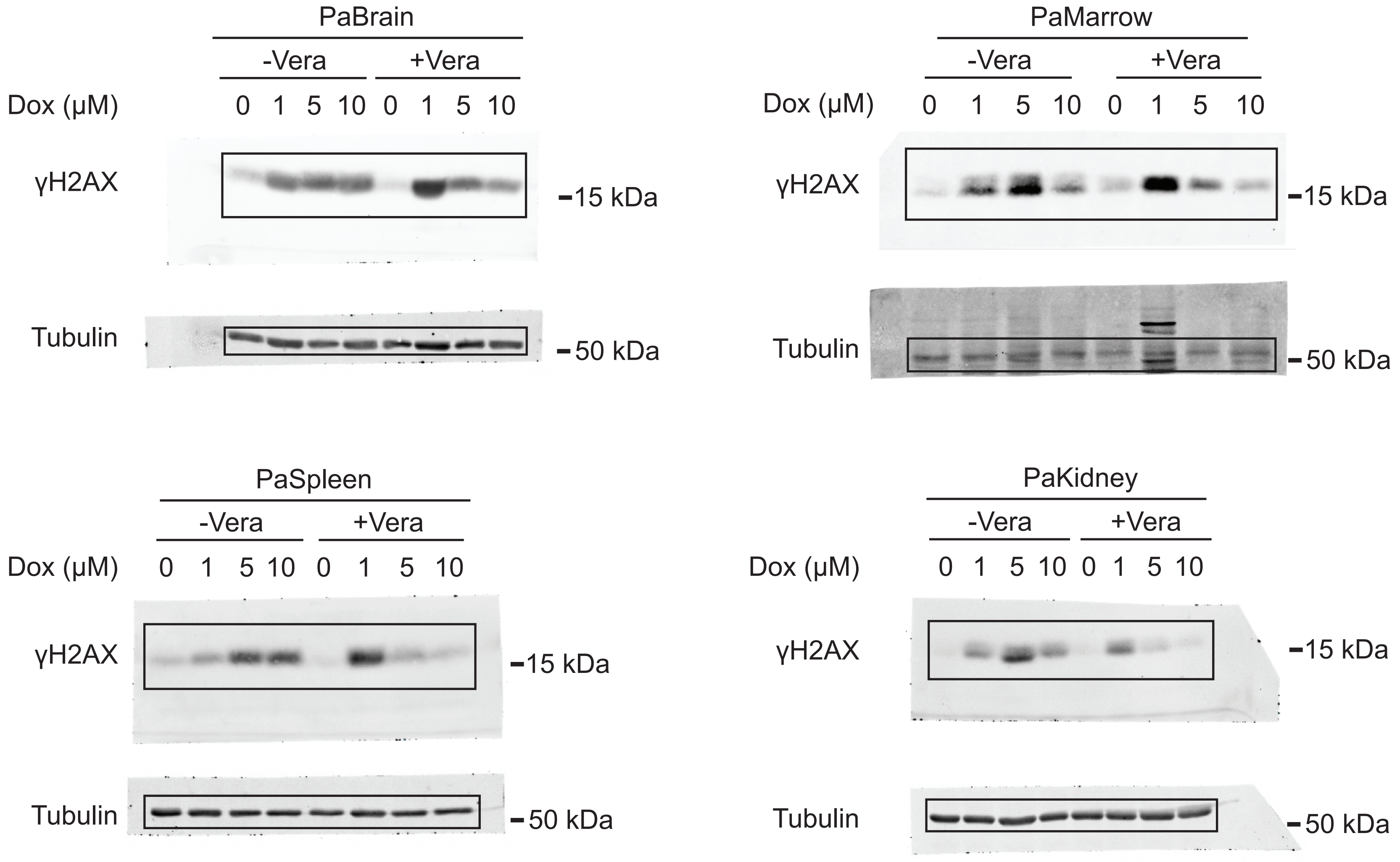

Supplementary Figure 7. Uncropped Western blots for Figure 4.

Figure 5D

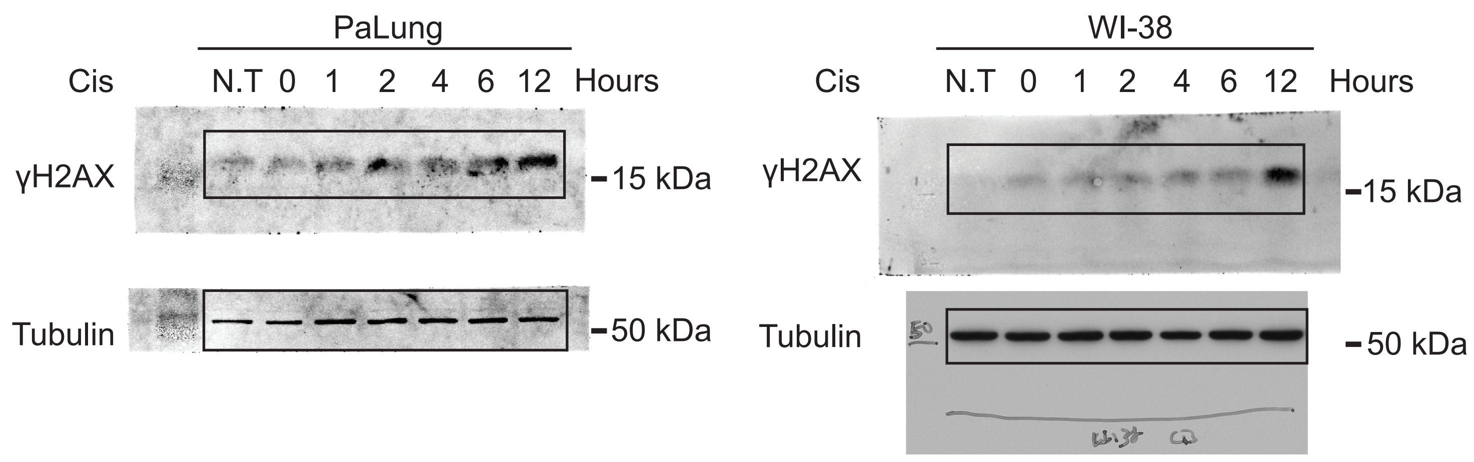

Supplementary Figure 8. Uncropped Western blots for Figure 5D.

Figure 6A

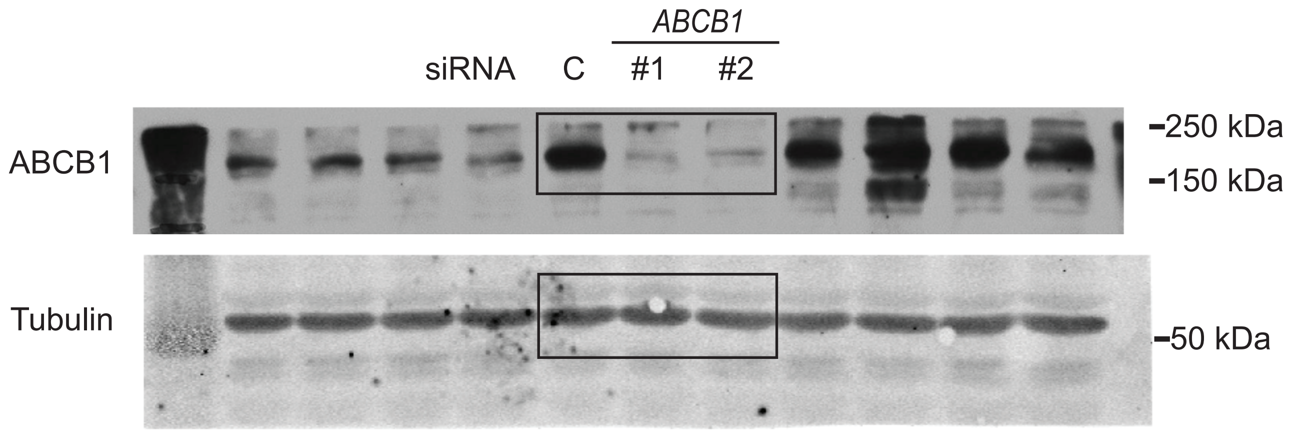

Supplementary Figure 9. Uncropped Western blots for Figure 6A.

Figure 7B

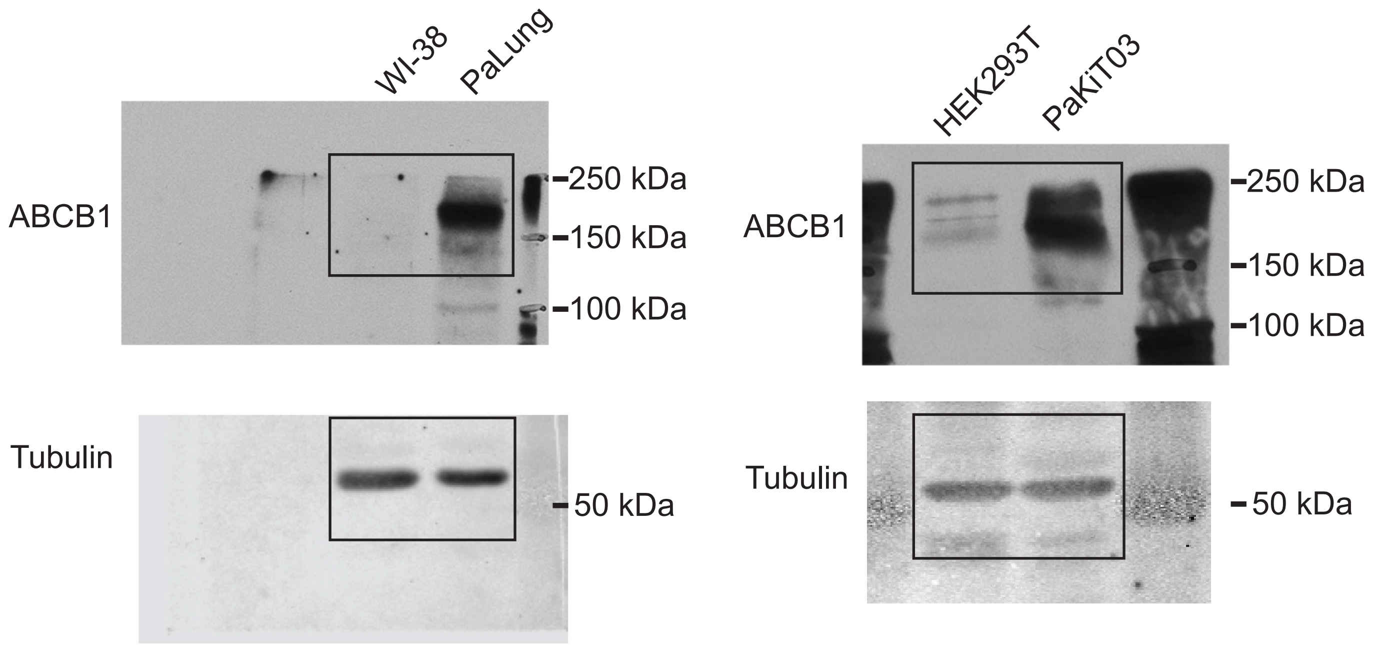

Figure 7D

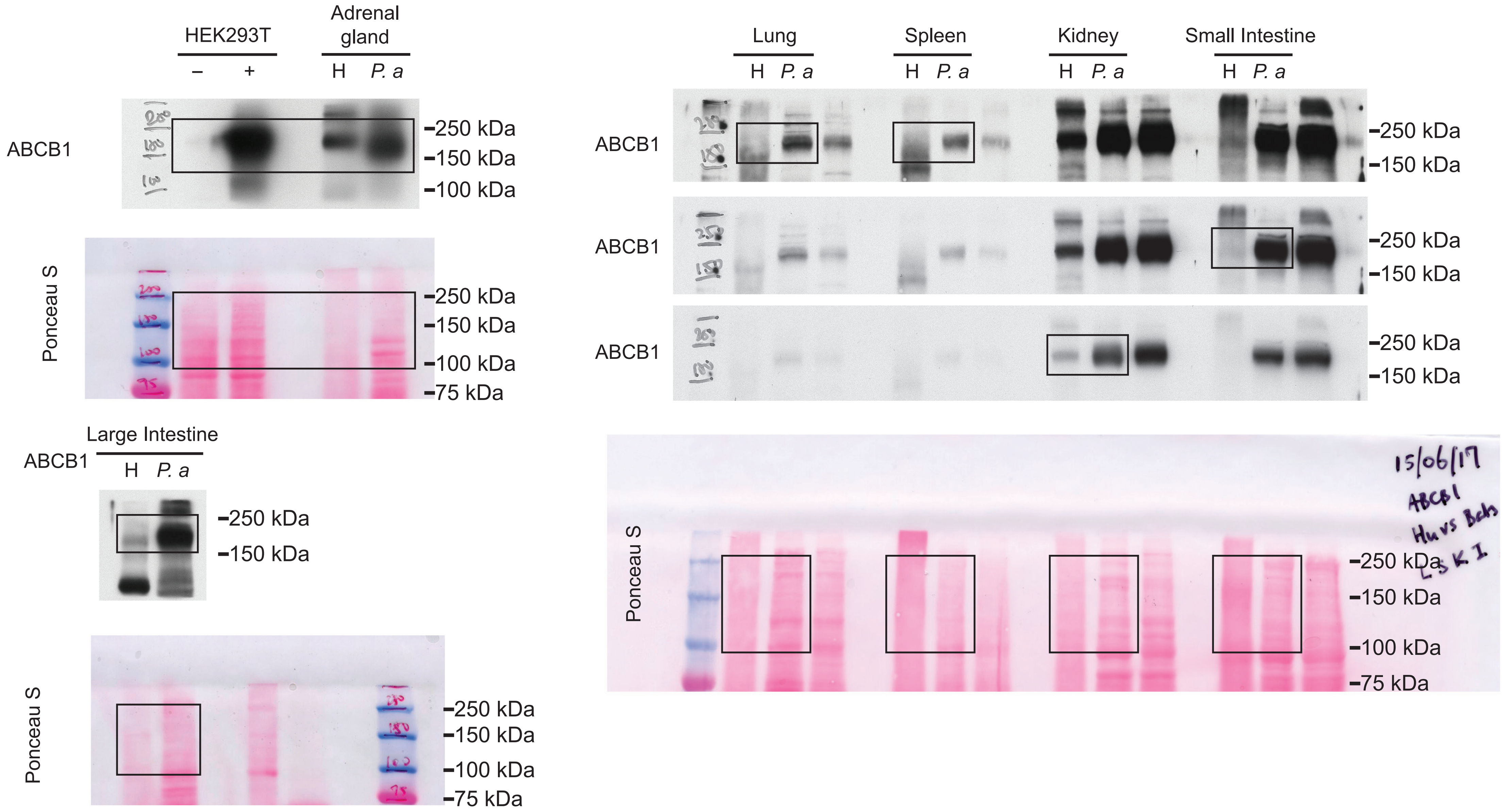

Supplementary Figure 10. Uncropped Western blots for Figure 7B and 7D.

Figure 7E

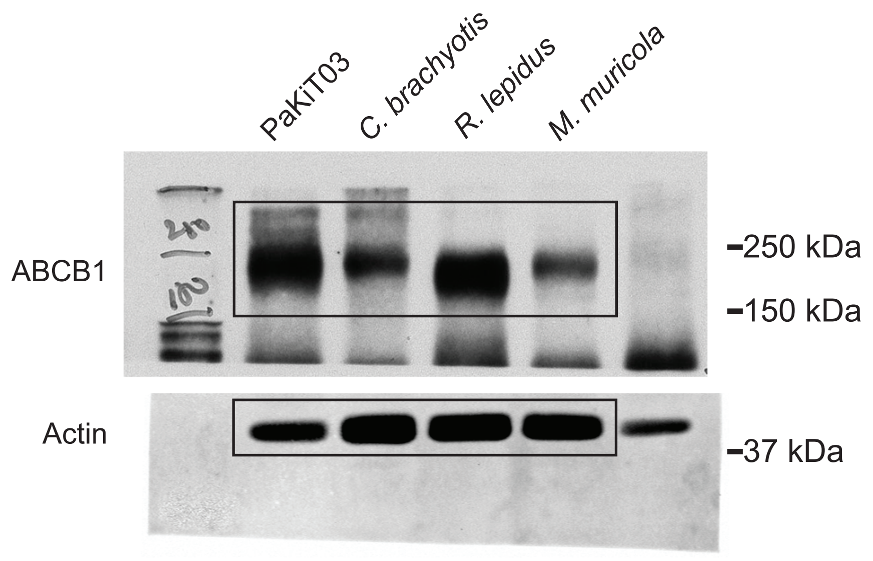

Figure 7G

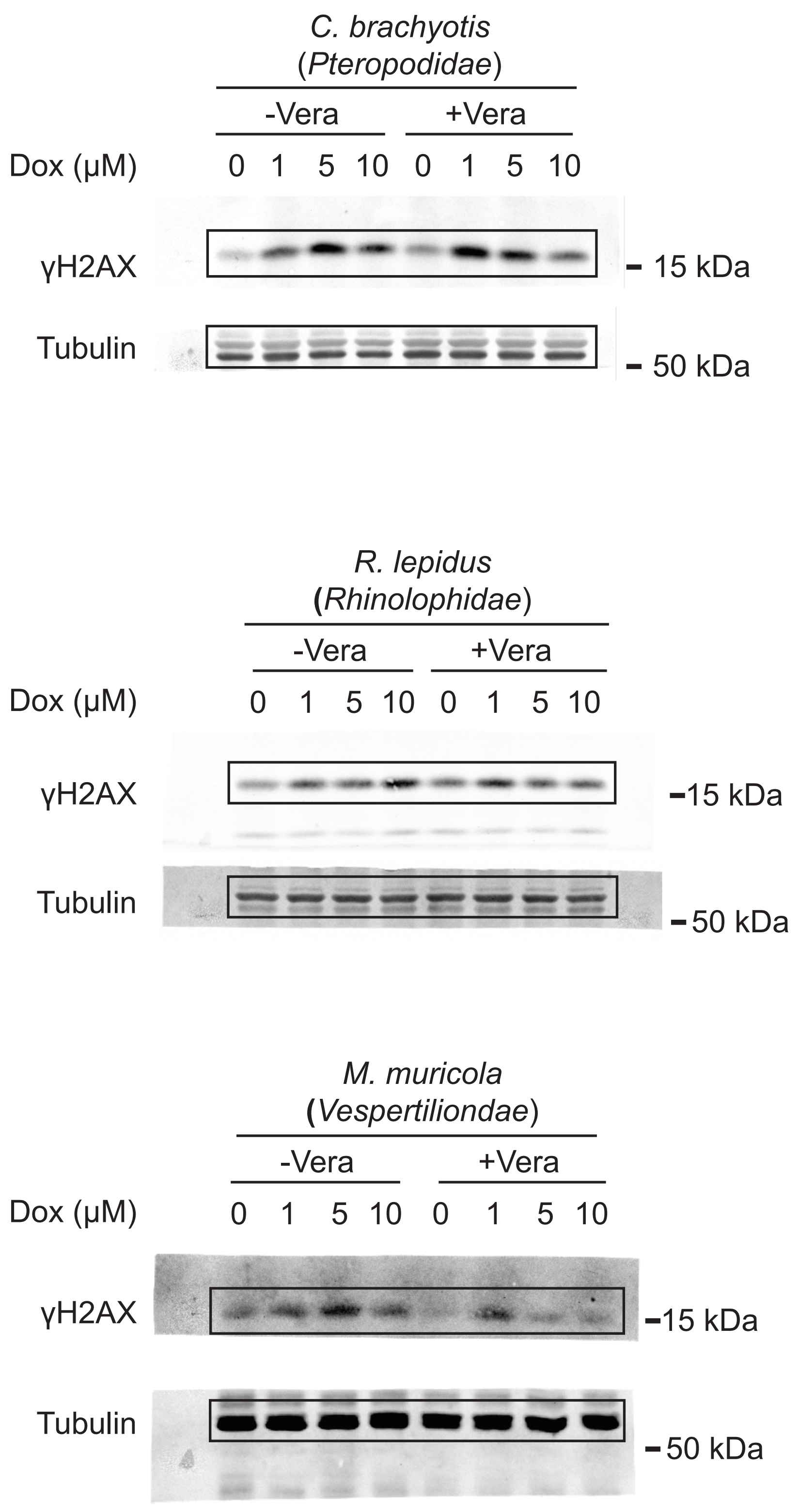

Supplementary Figure 11. Uncropped Western blots for Figure 7E and 7G.

Supplementary Figure 1A

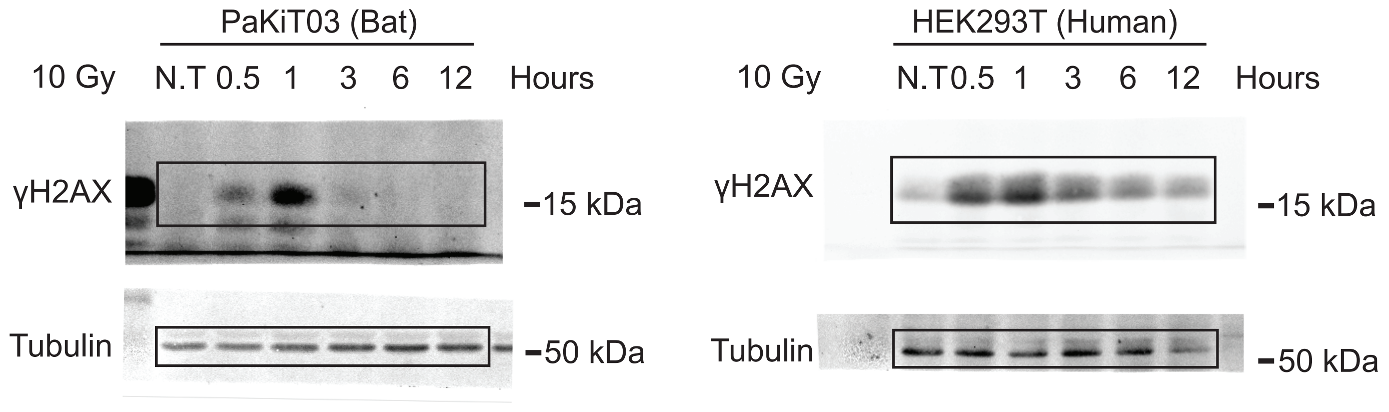

Supplementary Figure 1C

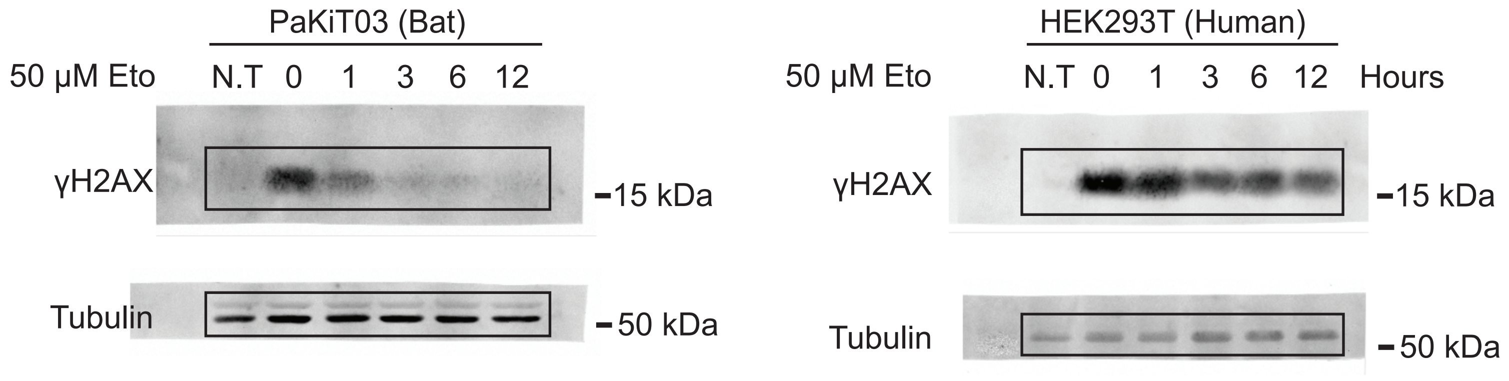

Supplementary Figure 12. Uncropped Western blots for Supplementary Figure 1A and 1C

Supplementary Figure 2A

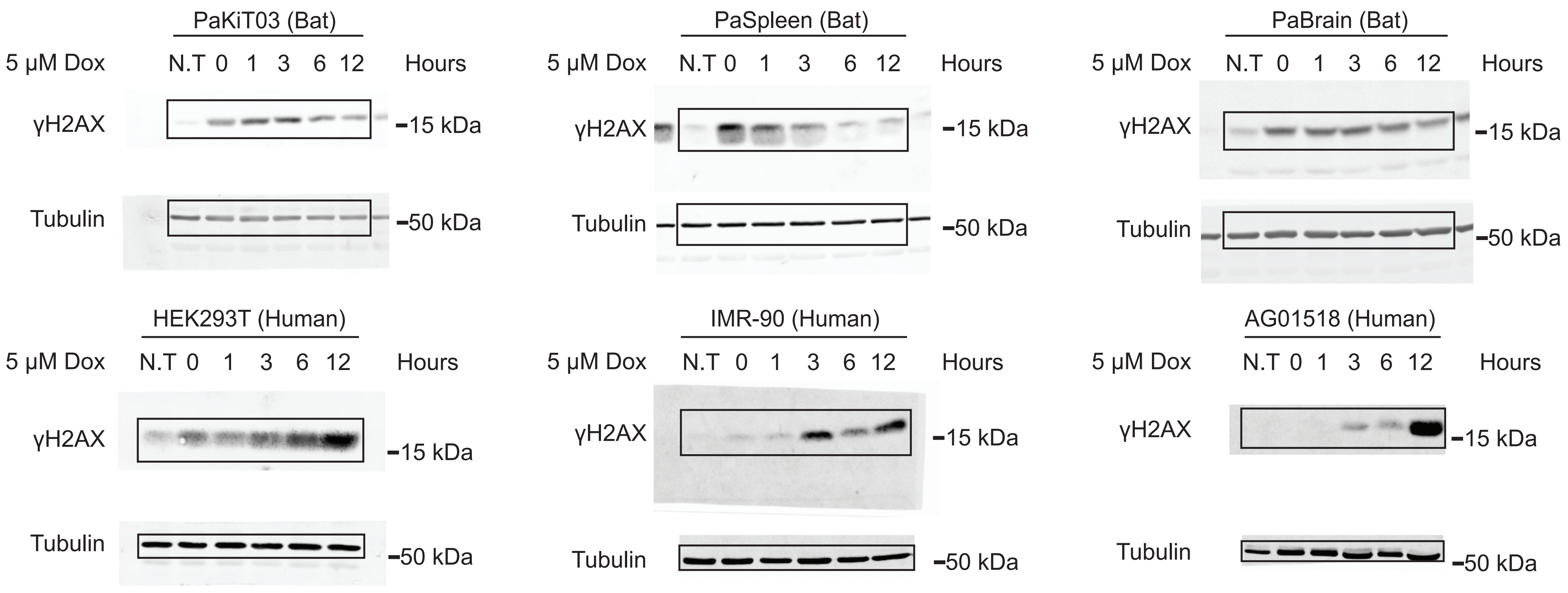

Supplementary Figure 13. Uncropped Western blots for Supplementary Figure 2A
