# Supplementary material for: ABCB1 protects bat cells from DNA damage induced by genotoxic compounds
Source: Nat Commun. 2019 Jun 27;10:2820. doi: 10.1038/s41467-019-10495-4 (PMC6597548; doi:10.1038/s41467-019-10495-4)
Supplement: Supplementary file 3 — Reporting Summary [file 41467_2019_10495_MOESM3_ESM.pdf]

nature research

Corresponding author(s): Koji Itahana

Last updated by author(s): May 30, 2019

Reporting Summary

Nature Research wishes to improve the reproducibility of the work that we publish. This form provides structure for consistency and transparency in reporting. For further information on Nature Research policies, see Authors & Referees and the Editorial Policy Checklist.

Statistics

For all statistical analyses, confirm that the following items are present in the figure legend, table legend, main text, or Methods section.

n/a	Confirmed	
☐	☒	The exact sample size (*n*) for each experimental group/condition, given as a discrete number and unit of measurement
☐	☒	A statement on whether measurements were taken from distinct samples or whether the same sample was measured repeatedly
☐	☒	The statistical test(s) used AND whether they are one- or two-sided<br>*Only common tests should be described solely by name; describe more complex techniques in the Methods section.*
☒	☐	A description of all covariates tested
☒	☐	A description of any assumptions or corrections, such as tests of normality and adjustment for multiple comparisons
☐	☒	A full description of the statistical parameters including central tendency (e.g. means) or other basic estimates (e.g. regression coefficient) AND variation (e.g. standard deviation) or associated estimates of uncertainty (e.g. confidence intervals)
☒	☐	For null hypothesis testing, the test statistic (e.g. *F*, *t*, *r*) with confidence intervals, effect sizes, degrees of freedom and *P* value noted<br>*Give P values as exact values whenever suitable.*
☒	☐	For Bayesian analysis, information on the choice of priors and Markov chain Monte Carlo settings
☒	☐	For hierarchical and complex designs, identification of the appropriate level for tests and full reporting of outcomes
☒	☐	Estimates of effect sizes (e.g. Cohen's *d*, Pearson's *r*), indicating how they were calculated

Our web collection on statistics for biologists contains articles on many of the points above.

Software and code

Policy information about availability of computer code

Data collection	LI-COR Odyssey (LI COR Biosciences), Olympus IX83 fluorescent microscope, Olympus IX71S1F3 fluorescent microscope, MACSQuant Analyzer 10 (Miltenyi Biotec), CFX96 System (BioRad), TECAN infinite M200
Data analysis	CASPlab software (Open source), CellProfilerTM software (Open source), BioRad CFX manager software (BioRad), Vector NTI Advance version 11.5 (Thermo Fisher Scientific), GraphPad Prism 7 (GraphPad), FlowJo (FlowJo LLC).

For manuscripts utilizing custom algorithms or software that are central to the research but not yet described in published literature, software must be made available to editors/reviewers. We strongly encourage code deposition in a community repository (e.g. GitHub). See the Nature Research guidelines for submitting code & software for further information.

Data

Policy information about availability of data

All manuscripts must include a data availability statement. This statement should provide the following information, where applicable:

- Accession codes, unique identifiers, or web links for publicly available datasets
- A list of figures that have associated raw data
- A description of any restrictions on data availability

Figure 5: accession [SRP044809], [https://www.ncbi.nlm.nih.gov/sra/SRX661189[accn]] and [https://www.ncbi.nlm.nih.gov/sra/SRX665117[accn]].
Supplementary Figure 4 and Table 1: Bos taurus – cow (XP_010802430.1), Canis lupus familiaris – dog (NP_001003215.2), Felis catus – cat (NP_001164535.1), Heterocephalus glaber – naked mole rat (XP_012934158.1), Homo sapiens - human (NP_000918.2), Macaca mulatta - Rhesus monkey (NP_001028059.1), Mus musculus - mouse ABCB1A (NP_035206.2) and mouse ABCB1B (NP_035205.1), Myotis davidii – David's myotis (XP_006779228.1), Pteropus alecto – black flying fox bat (XP_006925783), and Sus scrofa –pig (NP_001295175.1).
Supplementary Table 2: http://genomics.senescence.info/species/
All other data are available from the author upon reasonable request.

Field-specific reporting

Please select the one below that is the best fit for your research. If you are not sure, read the appropriate sections before making your selection.

☒ Life sciences ☐ Behavioural & social sciences ☐ Ecological, evolutionary & environmental sciences

For a reference copy of the document with all sections, see nature.com/documents/nr-reporting-summary-flat.pdf

Life sciences study design

All studies must disclose on these points even when the disclosure is negative.

Sample size	Sample number for each figure in this study are at least three and stated for each experiment in the figure legend.
Data exclusions	No data was excluded in any of the analysis of this study.
Replication	All experiments were conducted at least three times independently, unless stated differently in figure legends. Data collected from each experiment were reproducible with minimum standard deviations.
Randomization	No randomization techniques were used.
Blinding	No blinding method were used.

Reporting for specific materials, systems and methods

We require information from authors about some types of materials, experimental systems and methods used in many studies. Here, indicate whether each material, system or method listed is relevant to your study. If you are not sure if a list item applies to your research, read the appropriate section before selecting a response.

Materials & experimental systems

n/a	Involved in the study	
☐	☒	Antibodies
☐	☒	Eukaryotic cell lines
☒	☐	Palaeontology
☐	☒	Animals and other organisms
☒	☐	Human research participants
☒	☐	Clinical data

Methods

n/a	Involved in the study	
☒	☐	ChIP-seq
☐	☒	Flow cytometry
☒	☐	MRI-based neuroimaging

Antibodies

Antibodies used	γH2AX (05-636, Millipore, 1:3,000), tubulin (ab44928, Abcam, 1:5,000), actin (MAB1501, Millipore, 1:10,000), ABCB1 (sc-8313 H241, Santa Cruz, 1:1,000), 53BP1 (sc-22760, Santa Cruz, 1:250), HRP- conjugated secondary antibody (Thermo Scientific, 1:10,000), and fluorescence-abelled secondary antibodies (Thermo Scientific, 1:10,000 and Jackson ImmunoResearch, 1:100).
Validation	All antibodies used in this study were validated by the respective suppliers.

Eukaryotic cell lines

Policy information about cell lines

Cell line source(s)	Cell line sources fof all cell lines are listed in the Method section.
Authentication	All commercial cell lines were authenticated by the suppliers.<br>Primary cell lines derived from bats were not authenticated.
Mycoplasma contamination	All cell lines in this study were tested negative for mycoplasma.
Commonly misidentified lines (See ICLAC register)	WiDr cells

Animals and other organisms

Policy information about studies involving animals; ARRIVE guidelines recommended for reporting animal research

Laboratory animals	This study did not involve laboratory animals
Wild animals	Cynopterus brachyotis, Myotis muricola and Rhinolophus lepidus were captured in Singapore under the National Parks Board permit NP/RP11-011 and NP/RP14-109.
Field-collected samples	The study did not involve samples collected from the field.
Ethics oversight	National University of Singapore Institutional Animal Care and Use Committee (IACUC) Protocol B01/12

Note that full information on the approval of the study protocol must also be provided in the manuscript.

Flow Cytometry

Plots

Confirm that:

- [x] The axis labels state the marker and fluorochrome used (e.g. CD4-FITC).
- [x] The axis scales are clearly visible. Include numbers along axes only for bottom left plot of group (a 'group' is an analysis of identical markers).
- [x] All plots are contour plots with outliers or pseudocolor plots.
- [x] A numerical value for number of cells or percentage (with statistics) is provided.

Methodology

Sample preparation	Cells were pre-treated with 5 µM verapamil or cyclosporin A for 30 minutes, followed by incubation with doxorubicin for 3 hours. Cells were then trypsinized, washed withPBS once, and resuspended in PBS for analysis by MACSQuant Analyzer 10 (Miltenyi Biotec). Laser excitation and filter wavelengths were set at 488 nm and 614/50 nm respectively.
Instrument	MACSQuant Analyzer 10 (Miltenyi Biotec)
Software	FlowJo software
Cell population abundance	No cell sorting was performed.
Gating strategy	No extensive gating strategy were taken.

- [ ] Tick this box to confirm that a figure exemplifying the gating strategy is provided in the Supplementary Information.
