# Supplementary material for: ABCB1 protects bat cells from DNA damage induced by genotoxic compounds
Source: Nat Commun. 2019 Jun 27;10:2820. doi: 10.1038/s41467-019-10495-4 (PMC6597548; doi:10.1038/s41467-019-10495-4)
Supplement: Supplementary file 2 — Peer Review [file 41467_2019_10495_MOESM2_ESM.pdf]

Reviewers' comments:

Reviewer #1 (Remarks to the Author):

The hypothesis that the high expression of multidrug efflux pump ABCB1 contributes to support the longevity of bats is attractive. However, the evidence to show the direct involvement of ABCB1 in the longevity is poor. The conclusion that the expression of ABCB1 is higher than other animals is not supported by the results, either. The main result is just the comparison between cell lines. They also compared ABCB1 protein in various tissues from bat with that in human tissues (commercially available). Because ABCB1 is not expressed uniformly in human tissues, the comparison shown in Fig 7C is not convincing. The corresponding part of mouse and bat tissues should be used for the comparison. Because mouse tissues express two types of abcb1, the antibody (possibly C219) that recognizes both types should be used. The reasonable explanation why the high expression of ABCB1 supports the longevity of bats is also required.

Reviewer #2 (Remarks to the Author):

This article argues that high ABCB1 expression in bats protects them from chemical DNA damaging agents and may explain their relatively long lifespan. Using cell lines, the authors demonstrate that bat cells express higher levels of ABCB1 than cells from mice and humans, and accumulate higher levels of drugs that are known ABCB1 substrates, such as doxorubicin. Knockdown and chemical inhibition of the ABCB1 transporter sensitizes bat cells to killing with doxorubicin, and leads to an increase in DNA double strand breaks assessed directly by comet assays, and indirectly by gamma H2AX. This work represents a mechanism of resistance to DNA damage that is well known for cancer cells, but has not previously been described at the whole-organism level. Given the implications for longevity and cancer resistance, the paper will likely be of interest across several disciplines.
This study presents an exciting set of data that suggest the long lifespan and low cancer rates observed in bats may be explained by small molecule efflux, and not due to an increased DNA damage response. However the data do not support the strong statement in the discussion that "Our data refute alterations to the DDR pathway as a plausible mechanism for increased DNA damage resistance". In order to justify this central claim, a number of additional experiments and controls should be carried out. Even if the claim is softened, these experiments are needed to provide clarify the relative importance of DNA repair capacity and efflux as resistance mechanisms for bats exposed to DNA damaging agents.

1. This manuscript could be greatly strengthened by inclusion of additional methods for assessing DNA repair capacity. The authors rely heavily on gamma H2AX expression levels, which are an indirect measure of DNA double strand breaks. However the western blotting approach used here cannot distinguish gamma H2AX foci that are associated with double strand breaks from pan-nuclear gamma H2AX that can represent a preapoptotic signal (see for example Proc Natl Acad Sci U S A. 2010 Apr 13;107(15):6870-5)). Gamma H2AX also only reports on double strand breaks, but not other types of DNA damage. Importantly, doxorubicin may kill cells by inducing oxidative DNA damage, which may not be detected by the methods used in this manuscript.
2. The neutral comet assays employed in Fig. 6 also only report on double strand breaks, and were not used to confirm that rates of repair are similar between cells from bats versus cells from other mammalian organisms when the DNA damaging agent is not an ABCB1 substrate. For example, the relative rates of repair for DNA damage induced by ionizing radiation could be measured in human cells and bat cells. Comet assays should be performed at multiple time points to draw conclusions about DNA repair kinetics. An alkaline comet assay should be performed to measure repair of abasic sites and single strand breaks induced following exposure to reactive oxygen species. Inclusion of other types of DNA damaging agents, such as radiation and hydrogen peroxide, which induce oxidative damage independent of ABCB1 status would greatly strengthen the argument that bat cells

do not depend on increased DNA repair capacity to achieve relative resistance to DNA damaging agents.

3. The data in Figure 1C do not seem to be consistent with a mere increase in the intracellular concentration of doxorubicin as reported in Fig. 2. If one simply corrects the effective concentration for human WI-38 cells by multiplying by a factor of 2, the dose-dependence of gamma H2AX expression would still be different from that in human cells. This issue should be discussed further.

4. The data in Figure 1B are consistent with more rapid resolution of gamma H2AX in PaLung cells. Both WI-38 cells and PaLung cells have similar H2AX levels at t0. Once etoposide is removed, one would not expect new damage to occur for as long as 12 hours. Some discussion is needed with regard to why the data are not interpreted as reflective of more efficient repair in the PaLung cells.

5. A dose-response curve and an independent method of measuring DNA damage levels and repair kinetics are needed in addition to the data in Figure 1A to support the conclusion that there are no differences in repair capacity between the two cell types.

6. Many important experiments (Fig 1, Fig 2, Fig 4) are carried out using a single pair of cell lines (WI-38 and PaLung), or compare several bat cell lines only to WI-38. Data for at least one additional human cell line (ideally primary human cells from a different tissue) should be added to these experiments.

7. The absolute doxorubicin levels in Fig. 3D should be made available in a format analogous to that in Fig. 2A so the reader can assess the differences among bat, mouse and human cell lines in the absence of the verapamil.

8. A positive control for detection of human ABCB1 with the antibody used in the immunoblot presented in Fig. 7 is needed to confirm the absence (or low levels) of the protein in human tissues.
9. The manuscript could be greatly strengthened by an in vivo confirmation of the findings. Although the authors argue persuasively that longevity studies are not feasible with bats, an important test of the role of the ABCB1 transporter in tumorigenesis and tolerance of lethal viruses (Fig. 8) seems feasible by combining ABCB1 inhibition with the relevant exposures. An alternate, simpler in vivo test of the conclusion that bats uniquely resist DNA damaging agents by efflux mechanisms would be to measure LC50 values in bats and mice for a small molecule DNA damaging agent that is an ABCB1 substrate (e.g. doxorubicin) versus an efflux-independent agent (e.g. ionizing radiation).

Reviewer #3 (Remarks to the Author):

Koh et al., set out to determine if bats cells have better DNA damage response mechanisms than other mammals, which may underlie bats' unexpected longevity. This paper is an elegant, exquisite and in-depth exploration into the response of bat cells to genotoxic chemicals and shows that bats would be excellent models for cancer therapy studies. The exciting finding is that bat cells are able to remove genotoxic substances much faster than human or mouse cells through the ABCB1 transporters. Maybe this could explain the fact that cancer is almost never recorded in bats, not mentioned by the authors.

The authors are correct, this is one of the first studies of its kind, only possible because of the previous cell and genomic tools the authors have developed for bats. However, the authors have

oversold or misrepresented this study as a longevity study. It is not a longevity study as such. This is not where its novelty lies. This has lead to a lot of speculation and attempts to tie in bat immunity and potential adaptation to flight (particularly in the discussion), which perhaps is warranted but the data presented here do not show or support these speculations.

I recommend that this paper be resubmitted for another review, once rewritten with a different and more appropriate focus. While the mechanistic studies are exquisite and an example of what is required to establish novel model study species, the ecological and evolutionary aspects of bats presented in this paper are based on old literature that needs updating, sometimes is incorrect, misleading and needs a major rewrite. The results reported are important findings but they need to be correctly repackaged to reach their full impact and not mislead readers. Below are some of my major recommendations aimed to improve this paper and ensure that the bat biology, phylogenetic implications and interpretations are equally as excellent as these novel mechanistic studies.

>Page 3, Issues with bat aging data: First line. The authors indicate that the age range for bats is 10-40 years. Certain bat species live longer than 40 years. Myotis brandtii, with the oldest longevity record was at least 41 when re-caught, first captured as an adult of unknown age, considered at least >1 years of age given the fused finger bones, and re-caught 40 years later. They should use more up to date aging data for bat longevities, which would be AnAge < http://genomics.senescence.info/species/>. According to this website which reflects some of the most up to date literature, bats can live approx. 6-41 years, this reflect the range of longevities in bats Molossus molossus (>5.6 years)- Myotis brantii (>41 years). What they have not done is correct for body size, such as carried out in Austad 2010 J. Comp. Path V142, S10-S21 Fig 2. This is what sets bats apart from other species given their body size, not their actual chronological age, showing that Brandts bats can live up to 10 times longer than would be expected given their body size. This is what makes bats an interesting species to explore exceptional longevity and therefore has driven this study. Although they refer to Max Rubner's rate of living theory they really don't explore what this means in terms of making bats outliers. They need to highlight why bats are so unique given their longevity. This is not clear throughout.

> Using this rational Pteropus alecto is not an exceptionally long lived bat, with an longevity quotient of 1.67 as based on the data set of Healy et al 2014 Proc Roy Soc (DOI: 10.1098/rspb.2014.0298) analysed as in Austad 2010 J. Comp. Path V142, S10-S21 Fig 2. Our estimates for the other species are Cynoterus brachyotis 1.43; the average LQ for the genus Myotis is 4.108 (n=21); the average LQ for the genus Rhinolophus is 5.64 (n=2); Myotis brandtii is (8.23), humans (4.3).

Bats still do live longer than humans given their body size but not all species. They need to clarify the LQ for all the bats in their study to enable the readers get an estimate of where in the aging spectrum these species fall. They should also highlight that these data predominately come from field-based studies so may be an underestimation of true longevities, bats most likely live longer.

>Again the rationale for choice of study species must be described. I presume that one of the reasons for choosing these species is that the authors have already developed the appropriate cells lines and genomic data/tools that enable a study of this kind. This is a valid reason for choosing these taxa and this study does represent some of the first in-depth cellular functional studies in bats but it needs to be made clear. Of the ~1300 species of bats why did they choose Pteropus alecto, Myotis davidii etc. This needs more explanation.

>Page 3 Reference to enzymatic assessment of free radical scavenging as ref 14 is incorrect, this is the field record for Myotis brandtii longevity, rather than ref 31, which I presume they meant to refer too.

>Fruitvore such be Frugivore throughout.

>Page 4. The authors state definitively that bats and humans are similarly responsive to DNA damage as induced by irradiation (Fig 1 A). Now I'm intrigued why they did not also test mouse cells here? Both human and bats respond in the same manner but bats should respond more like mice given their body size and metabolism, according to the logic used in this paper. The fact that they respond more similar to human indicates that they are responding more like a large long lived mammal rather than a small short lived mammal as would be expected. Again this is why I feel the underlying rationale for using bats as a model species for aging resistance needs to be better explained throughout.

>Page 4. One single part to one figure (Fig 1A) and one experiment is not enough to definitively state that bat's DNA damage response is not driving their unique longevities. Either more data need to be provided or this needs to be toned down (see comments below).

> Page 9. The authors have bat cell lines derived from other bat species, why did they only include these species in the drug efflux comparison (Fig 3D, 3E) and not in all of the other studies? I presume that this is because of the limited resources of the material available from other species but it needs to be highlighted. I would have liked to have seen how the different bat species responded to the different DNA damage experiments. I however understand that this may not be possible, but given the different LQs of these species it would be interesting.

>The fact that these diverse bat species are only included in one experiment does not necessarily warrant a conclusion or statement from the authors that these findings are based on a wide phylogenetic study and one of the first of its kind. Yes it is one of the first mechanistic studies of its kind but it is not based on deep phylogenetic representation from bats for all experiments. This needs further clarification.

>The authors are correct in the efflux experiment, they do sample the basal bat divergences but what they are comparing are the response of wild outbred species' recently derived cell lines to experimental mice and over passaged human cells. Are they not concerned the differential response they see in bats compared with humans and mice is nothing more than a wild versus captive/derived response? Lab mice react in completely different fashion to their wild caught sister-species when faced with the same experimental conditions. It would be great to have a non-bat wild derived cell line, something that lives shorter or what is expected for its body size (e.g. shrew, wild mice). I'm not sure that this is a feasible request given how long it would take to establish these resources. The authors should indicate that this could be a problem, likely or unlikely in the manuscript.

>What is regulating the expression of the ABCB1 in bats? This is an interesting question. miRNAs have been suggested as a possible regulatory mechanism that may be driving extended longevity in bats (Huang et al , BMC genomics 17 (1), 906) this should be included somewhere in the text.

> Discussion. The authors cannot state that based on their experiments that they refute the DDR pathway as a plausible mechanism for increased damage resistance. This is a little misleading and overarching as they only do one experiment on one bat species (Fig1A). They also seem to have a different interpretation of the findings of Podlutsky et al ref 31. The fact that bat cells respond like primate cells and not like mice cells is unexpected and shows that perhaps DDR in bats is unique given their body size. Also according to Podlutsky et al ref 31, humans have better DDR than mice , as do bats for BER, but not NER pathways. This needs further exploration.

> Discussion. They do not study multiple phylogenetically distant bats for all experiment just one. Again this is misleading.

>Discussion. Why would bats be faced with more vulnerability to a wide spectrum on xenobiotics? Assuming, given the claims the authors make, that this adaptation of removing genotoxic substances is ancestral, the bat ancestor would not have been faced with such a large range of different environments. Bats diverged into their different biological niches over millions of years (Teeling et al,

Science 307 (5709), 580-584). This rationale either needs to be removed or better qualified.
>Discussion. Bats don't experience the majority of these toxic substances in the wild. The authors refer to cadmium as a naturally occurring substance. How often to bats experience this toxin? Again I'm not sure what selective pressure would have driven this adaptation in the bat ancestor? The evolution of the ABCB1 expression should predate the massive expansion into all these niches, a pre-adaptation which has allowed bats to expand? But then why did it evolve in the first place? Again the authors must reconsider and further explore this rationale.

> Discussion. The authors need to update their references and value pertaining to the metabolic consequences of flight in bats. There have been many more recent studies on this area.

> Discussion: The authors cannot state that they have shown in their genome paper (40) that bats have adaptively lost PYHINS to reduce inflammation as an adaptation to flight. This is a gross over statement of what their paper showed, which was simply a lack of PYHINS in bat s compared with other mammals which they correlated with flight acquisition. This really is misleading. Also the tenuous link between ABCB1 and anti-inflammation/immunity mechanisms needs to be clarified. It might be correct but there are no data to show this here. I think reporting their new findings in light of these other speculated adaptations is a little contrived. I'm not sure that this brings anything more to this paper and may even dilute the results.

>Discussion. I really commend them on their explanations about why it is so difficult to do any in vivo studies on bats. This is important and further highlights the utility of their system.

>Discussion. Their final statement again is really speculative, Fig 8 again is suggestive. The data don't show this. They need to refer to their real results- bats are better able to remove genotoxic substances from their cells better than mice and humans and this indicates that they could be a great model to study anti-cancer mechanisms. Anything else is speculation and if they want to include it then they need to specify that this is just speculative and suggestive.

> They need to update the literature on aging studies and genomic studies in bats (e.g. Siem et al. Nature Communications 2013, Myotis brandtii genome) and other outlier species naked mole rat, not only self-citing. Some key literature are hi

Response to the reviewers

Reviewer #1

The hypothesis that the high expression of multidrug efflux pump ABCB1 contributes to support the longevity of bats is attractive. However, the evidence to show the direct involvement of ABCB1 in the longevity is poor.

Response: We would like to thank the reviewer for the constructive suggestions. We agree that our results do not directly show the involvement of ABCB1 in longevity. Establishing an *in vivo* bat model to study the molecular mechanisms of longevity is challenging since bats have a long lifespan and low reproduction rates (Wang, L.F., *et al*. 2011). Therefore, given the overall comments of the reviewer and as suggested by Reviewer #3, we decided to rewrite the manuscript to change the focus to DNA damage resistance via efflux of genotoxic substances as a potentially novel tumour suppressive mechanism in bats. DNA damage is well-established as the major cause of tumours (Polo, S. E. and Jackson, S. P. 2011; Tubbs, A. and Nussenzweig, A. 2017). Reviewers #2 and #3 both mentioned that the strength and novelty of our manuscript is the finding that efficient efflux mediated by ABCB1 reduces DNA damage in bat cells. We hope that the reviewer agrees with the altered emphasis.

Polo, S. E., Jackson S. P. Dynamics of DNA damage response proteins at DNA breaks: a focus on protein modifications. Genes Dev ***25****, 409-433 (2011).*

Tubbs, A., Nussenzweig A. Endogenous DNA Damage as a Source of Genomic Instability in Cancer. Cell ***168****, 644-656 (2017).*

Wang, L.F., Walker P. J., Poon L. L. M. Mass extinctions, biodiversity and mitochondrial function: are bats 'special'as reservoirs for emerging viruses? Curr Opin Virol ***1****, 649-657 (2011).*

The conclusion that the expression of ABCB1 is higher than other animals is not supported by the results, either. The main result is just the comparison between cell lines. They also compared ABCB1 protein in various tissues from bat with that in human tissues (commercially available). Because ABCB1 is not expressed uniformly in human tissues, the comparison shown in Fig 7C is not convincing. The corresponding part of mouse and bat tissues should be used for the comparison. Because mouse tissues express two types of abcb1, the antibody (possibly C219) that recognizes both types should be used.

Response: We appreciate the reviewer to point out the over-interpretation of the data. We agree that our previous data were not sufficient to support the conclusion that ABCB1 expression is higher in bats than in other mammals. To make such a statement, it is required to assess the ABCB1 expression in various tissues from many different mammals. Adding the ABCB1 expression profile of mice is obviously not strong enough to make such a conclusion. Unfortunately, it is challenging for us to obtain a variety of tissues from multiple other mammals and assess ABCB1 expression because of the strict animal regulations in Singapore. In addition, the current commercial ABCB1 antibodies might have different affinities for ABCB1 in each mammalian species, which would obscure a comparison of ABCB1 protein levels between different species. Therefore, we removed this statement from the revised manuscript.

We chose to strengthen the point that bats have broad and higher expression of ABCB1 compared to human, and therefore added two more tissues from bat and human (Figure 7D). We used the ABCB1 antibody clone H241, which was raised against human ABCB1. Since the epitope sequence detected by the H241 antibody is not 100% conserved between human and bat (92% similarity between human and bat), this antibody might have a lower affinity to bat ABCB1 than to human ABCB1. Thus, the H241 antibody might underestimate the levels of ABCB1 in bat, compared to human ABCB1. Nevertheless, we confirmed the specificity of the H241 antibody for bat ABCB1, as *ABCB1* knockdown efficiently depleted the protein (Fig. 6A). We also included new RT-qPCR data that compare *ABCB1* mRNA levels in different tissues from bat and human to support the results of our Western blotting analysis (Fig. 7C). We would like to emphasize that we did compare the expression of ABCB1 using the same origin of tissues between human and bat since ABCB1 is not expressed uniformly in human tissues. We examined the tissues that are known to highly express ABCB1 in human such as kidney, small intestine, large intestine, and adrenal gland, as well as the tissues which are known to express very low or no ABCB1 in human such as lung and spleen (Fig. 7D). We hope that the reviewer agrees that we fairly compared the expression of ABCB1 between bat and human and finds the results in the revision convincing.

Although the revision shows a comparison of ABCB1 expression between bat and human only, we did examine ABCB1 levels in mouse tissues by Western blotting, using ABCB1 antibodies clone C219 (as suggested by the reviewer) and clone H241 (the one we used in the initial submission and the revision). Both clones C219 and H241 are well-established for their ability to detect the two forms of mouse ABCB1 by Western blotting (Katoh, M., *et al*. 2006; Boston-Howes, W., *et al*. 2008; Kim, I. W., *et al*. 2008).

First, we compared the sensitivities of the C219 and H241 antibodies using HEK293T human cell lines transfected with a human ABCB1 expressing plasmid. In our hands, the H241 clone showed a similar or even better sensitivity than the C219 clone toward human ABCB1 protein (Figure A, below). We then used the two antibodies to examine protein lysates extracted from lungs derived from human, bat and mouse. Unfortunately, both antibodies generated substantial non-specific signals in mouse tissue lysates that interfered with the detection of mouse ABCB1 (Figure B, below). Therefore, we were unable to compare the levels of ABCB1 protein between bat, human, and mouse in tissues. However, it was reported that the phenotypes of mice deleted for *Abcb1a* alone or both *Abcb1a* and *Abcb1b* are limited to the tissues that are known to express ABCB1 in human, such as brain, kidney, intestine and liver (Schinkel, A. H., *et al*. 1997; Schinkel, A. H., *et al*. 1994; Croop, J. M., *et al*. 1989). These data suggest that the functions of ABCB1a and ABCB1b in mice are limited to the same tissues as in human.

Nevertheless, we thank the reviewer for the suggestion of using another species (mouse) to strengthen our conclusions that bat cells have a better efflux capability than human and

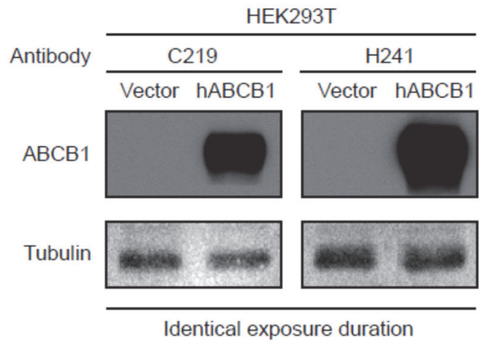

Figure A: Comparison of ABCB1 antibodies clone C219 and clone H241. HEK293T cells were transfected with a human ABCB1 expressing plasmid. Proteins were extracted 2 days after transfection and analysed by Western blotting. Tubulin was used as a loading control. Films of each protein were exposed for the same duration.

mouse cells, which leads to reduced DNA damage. We now added MEFs (mouse embryonic fibroblasts) to the majority of our functional assays to examine the DNA damage and doxorubicin efflux activity (Fig. 1, 2, 3A, 3B, 4A, 4B, and 7H).

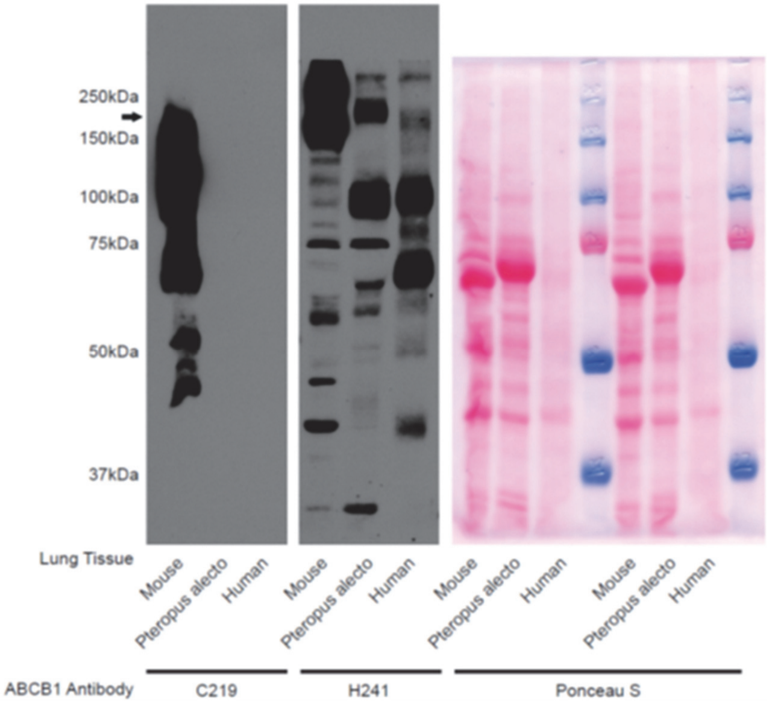

Figure B: Western blotting analysis of ABCB1 in lung tissues derived from different mammal species. Protein were extracted from lung tissues of the indicated species. Ponceau S staining is served as a loading control. The arrow indicates the position of ABCB1 protein.

Boston-Howes, W., Williams, E. O., Bogush, A., Scolere, M., Pasinelli, P., Trotti, D. Nordihydroguaiaretic acid increases glutamate uptake in vitro and in vivo: therapeutic implications for amyotrophic lateral sclerosis. Exp Neurol ***213****, 229-237 (2008).*

Croop, J. M., et al. The three mouse multidrug resistance (mdr) genes are expressed in a tissue-specific manner in normal mouse tissues. Mol Cell Biol ***9****, 1346-1350 (1989).*

Katoh, M., Suzuyama, N., Takeuchi, T., Yoshitomi, S., Asahi, S., Yokoi, T. Kinetic analyses for species differences in P-glycoprotein-mediated drug transport. J Pharm Sci ***95****, 2673-2683 (2006).*

Kim, I. W., Booth-Genthe, C., Ambudkar, S. V. Relationship between drugs and functional activity of various mammalian P-glycoproteins (ABCB1). Mini Rev Med Chem ***8****, 193-200 (2008).*

Schinkel, A. H., et al. Disruption of the mouse mdr1a P-glycoprotein gene leads to a deficiency in the blood-brain barrier and to increased sensitivity to drugs. Cell 77, 491-502 (1994).

Schinkel, A. H., et al. Normal viability and altered pharmacokinetics in mice lacking mdr1-type (drug-transporting) P-glycoproteins. Proc Natl Acad Sci U S A ***94****, 4028-4033 (1997).*

The reasonable explanation why the high expression of ABCB1 supports the longevity of bats is also required.

Response: As mentioned above, we agree that this aspect of the manuscript was speculative and no longer discuss longevity in the revision. We rewrote the manuscript to focus on the improved ABCB1-mediated efflux in bat, leading to reduced DNA damage.

Reviewer #2:

This article argues that high ABCB1 expression in bats protects them from chemical DNA damaging agents and may explain their relatively long lifespan. Using cell lines, the authors demonstrate that bat cells express higher levels of ABCB1 than cells from mice and humans, and accumulate higher levels of drugs that are known ABCB1 substrates, such as doxorubicin. Knockdown and chemical inhibition of the ABCB1 transporter sensitizes bat cells to killing with doxorubicin, and leads to an increase in DNA double strand breaks assessed directly by comet assays, and indirectly by gamma H2AX. This work represents a mechanism of resistance to DNA damage that is well known for cancer cells, but has not previously been described at the whole-organism level. Given the implications for longevity and cancer resistance, the paper will likely be of interest across several disciplines.

This study presents an exciting set of data that suggest the long lifespan and low cancer rates observed in bats may be explained by small molecule efflux, and not due to an increased DNA damage response. However the data do not support the strong statement in the discussion that "Our data refute alterations to the DDR pathway as a plausible mechanism for increased DNA damage resistance". In order to justify this central claim, a number of additional experiments and controls should be carried out. Even if the claim is softened, these experiments are needed to provide clarify the relative importance of DNA repair capacity and efflux as resistance mechanisms for bats exposed to DNA damaging agents.

We thank for the reviewer for recognising the significance of our work and for the comments.

1. This manuscript could be greatly strengthened by inclusion of additional methods for assessing DNA repair capacity. The authors rely heavily on gamma H2AX expression levels, which are an indirect measure of DNA double strand breaks. However the western blotting approach used here cannot distinguish gamma H2AX foci that are associated with double strand breaks from pan-nuclear gamma H2AX that can represent a preapoptotic signal (see for example Proc Natl Acad Sci U S A. 2010 Apr 13;107(15):6870-5)). Gamma H2AX also only reports on double strand breaks, but not other types of DNA damage. Importantly, doxorubicin may kill cells by inducing oxidative DNA damage, which may not be detected by the methods used in this manuscript.

Response: We sincerely thank the reviewer for the recommendations and guidance. We agree with the reviewer that we cannot distinguish γH2AX foci associated with double strand breaks (DSBs) from pan-nuclear γH2AX by Western blotting analysis. To address this reviewer's concern, we performed immunofluorescence staining to detect γH2AX at multiple time points after treatment with γ-irradiation or doxorubicin using WI-38, MEF, and PaLung cells. We found that most cells displayed γH2AX foci rather than pan-nuclear γH2AX (Supplementary Fig. 1B and 2B), suggesting that the elevated γH2AX observed by Western blotting analysis mainly reflects an association with DSBs.

To address the reviewer's request for an additional method to assess DNA repair capacity, we added immunofluorescence staining of the DNA repair protein 53BP1, another

widely-used DNA damage marker (Panier, S. and Boulton, S. J. 2014; Croco, E.*, et al.* 2017). Our new data (Fig. 1B and 2C) support the changes in γH2AX observed by Western blotting analysis (Fig. 1A and 2B).

As the reviewer mentioned, doxorubicin can induce DNA damage via multiple mechanisms, which include DNA intercalating to inhibit topoisomerase II activity, histone eviction, and induction of oxidative stress to cause single and double strand DNA breaks (Pang, B., *et al*. 2013; Yang, F., *et al.* 2014). The mechanisms of cell death induced by doxorubicin are cell context-dependent and could be mediated by DNA damage or be independent from DNA damage such as replication stress or oxidative stress to proteins and other organelles (Yang, F., *et al.* 2014).

Importantly, our data show that bat cells accumulate less doxorubicin compared to human and mouse cells, leading to reduced DSBs, as monitored by changes in γH2AX and 53BP1, and that ABCB1 is responsible for the reduced doxorubicin accumulation and less DNA damages in bat cells. Given the complex effects of doxorubicin, we rewrote the manuscript and simplified our point by focusing on the ability of ABCB1 to suppress the accumulation of intracellular doxorubicin, and thus prevent DNA damage and cell death.

Croco, E., et al. DNA Damage Detection by 53BP1: Relationship to Species Longevity. J Gerontol A Biol Sci Med Sci ***72****, 763-770 (2017).*

Pang, B., et al. Drug-induced histone eviction from open chromatin contributes to the chemotherapeutic effects of doxorubicin. Nat Commun ***4****, 1908 (2013).*

Panier, S., Boulton, S. J. Double-strand break repair: 53BP1 comes into focus. Nat Rev Mol Cell Biol ***15****, 7-18 (2014).*

Yang, F., Teves, S. S., Kemp, C. J., Henikoff, S. Doxorubicin, DNA torsion, and chromatin dynamics. Biochim Biophys Acta ***1845****, 84-89 (2014).*

2. The neutral comet assays employed in Fig. 6 also only report on double strand breaks, and were not used to confirm that rates of repair are similar between cells from bats versus cells from other mammalian organisms when the DNA damaging agent is not an ABCB1 substrate. For example, the relative rates of repair for DNA damage induced by ionizing radiation could be measured in human cells and bat cells. Comet assays should be performed at multiple time points to draw conclusions about DNA repair kinetics. An alkaline comet assay should be performed to measure repair of abasic sites and single strand breaks induced following exposure to reactive oxygen species. Inclusion of other types of DNA damaging agents, such as radiation and hydrogen peroxide, which induce oxidative damage independent of ABCB1 status would greatly strengthen the argument that bat cells do not depend on increased DNA repair capacity to achieve relative resistance to DNA damaging agents.

Response: We would like to thank the reviewer for these suggestions, which we reply to below. To compare the DNA repair rate independent of ABCB1 between different species, we performed immunofluorescence staining of 53BP1 after γ-irradiation. γ-irradiation should deliver an equal amount of stress to the cells derived from the different species independently of ABCB1. As mentioned above, 53BP1 is a widely-used marker to assess DNA DSBs in various mammalian species (Panier, S. and Boulton, S. J. 2014; Croco, E.*, et al.* 2017).

We quantified the number of 53BP1 foci at the different time points after γ-irradiation and inferred the amount of DNA damage and the rate of the DNA repair in bat and human cells. We also included mouse cells for the comparison in the revised manuscript. The number of

53BP1 foci was similarly increased in bat and human cell lines exposed to γ-irradiation and similarly reduced over time (Fig. 1B), suggesting that their repair rate is similar. On the other hand, and consistent with a previous report of DNA repair (Cortopassi, G. A. and Wang, E. 1996), mouse cells responded to γ-irradiation more slowly, accumulating 53BP1 foci over time after treatment (Fig. 1B). Given these findings and that we performed the assay under only one condition (10 Gy of γ-irradiation), we agree that our findings do not "refute alterations to the DDR pathway as a plausible mechanism for increased DNA damage resistance". We removed this claim to avoid over-interpreting our results and misleading readers. Cells likely have multiple mechanisms to prevent DNA damage, and our data show that ABCB1-mediated efflux is a novel mechanism that protects bat cells from DNA damage.

As the reviewer suggested, an alkaline comet assay would provide valuable information on the total amount of DNA damage, which include single strand breaks (SSBs), DSBs, and abasic sites (Pu, X., *et al.* 2015). Neutral comet assay also detects not only DSBs, but also SSBs. It is difficult to distinguish between DSBs and SSBs by alkaline and neutral comet assay (*Collins, A. R., et al. 2008*). Thus, we utilized the neutral comet assay to detect the amount of DNA damages regardless of DSBs or SSBs (Fig. 6).

Collins, A. R., et al. The comet assay: topical issues. Mutagenesis 23, 143-151 (2008).

Cortopassi, G. A., Wang, E. There is substantial agreement among interspecies estimates of DNA repair activity. Mech Ageing Dev ***91****, 211-218 (1996).*

Croco, E., et al. DNA Damage Detection by 53BP1: Relationship to Species Longevity. J Gerontol A Biol Sci Med Sci ***72****, 763-770 (2017).*

Panier, S., Boulton, S. J. Double-strand break repair: 53BP1 comes into focus. Nat Rev Mol Cell Biol ***15****, 7-18 (2014).*

Pu, X., Wang, Z., Klaunig, J. E. Alkaline Comet Assay for Assessing DNA Damage in Individual Cells. Curr Protoc Toxicol ***65****, 3 12 11-11 (2015).*

3. The data in Figure 1C do not seem to be consistent with a mere increase in the intracellular concentration of doxorubicin as reported in Fig. 2. If one simply corrects the effective concentration for human WI-38 cells by multiplying by a factor of 2, the dose-dependence of gamma H2AX expression would still be different from that in human cells. This issue should be discussed further.

Response: We appreciate the reviewer's comments. We agree that based on the results from the previous Fig. 2, the amount of doxorubicin accumulated in human WI-38 cells was only about 2 times higher than in bat PaLung cells, which does not fully explain the difference in dose-dependence of γH2AX between bat and human cells (new Fig. 2A and 2E). One possible explanation is the efficiency of histone eviction. The threshold of doxorubicin required to trigger histone eviction might be higher for bat cells than human cells. Unfortunately, there is no literature addressing the efficiency of histone eviction in bat cells. Although we are not sure what accounts for this phenomenon, we demonstrated that the inhibiting ABCB1 efflux in bat cells was sufficient to trigger an accumulation of doxorubicin (Fig. 3, 5C, 7F, and 7H, and Supplementary Fig. 3) and doxorubicin dose-dependent histone eviction (Fig. 4), suggesting that the expression and activity of ABCB1 in bat cells contributes to the distinct levels of γH2AX.

4. The data in Figure 1B are consistent with more rapid resolution of gamma H2AX in PaLung cells. Both WI-38 cells and PaLung cells have similar H2AX levels at t0. Once etoposide is removed, one would not expect new damage to occur for as long as 12 hours. Some discussion is needed with regard to why the data are not interpreted as reflective of more efficient repair in the PaLung cells.

Response: We thank the reviewer for raising this point. We think that residual intracellular etoposide remains in cells for a while even after it is removed from the culture media, which could induce further DNA damage, as similar to residual doxorubicin whose red fluorescence remains for some time after its removal from media. We expect that the concentration of residual intracellular etoposide in WI-38 cells is higher than in PaLung cells, since etoposide is a well-known substrate of ABCB1. Therefore, the patterns of γH2AX could reflect not only the rate of DNA repair, but also the DNA damage caused by residual intracellular etoposide. It is difficult to measure the levels of residual intracellular etoposide in each cell line at each time point, because etoposide does not have auto-fluorescence, unlike doxorubicin. Therefore, it is difficult to determine to what extent differential repair rates or residual etoposide-induced DNA damage contribute to the distinct resolution of γH2AX in WI-38 vs. PaLung cells. We cannot exclude the possibility that PaLung cells may have a superior mechanism to repair DNA damage toward etoposide. Therefore, from the data in Fig. 1C and Supplementary Fig. 1C, we simply conclude that bat cells are more resistant than human cells to etoposide-induced DNA damage. We elaborate on the issue of the difficultly to compare the repair rate of DNA damage induced by the substrates of ABCB1 in the Discussion (line 406-417).

5. A dose-response curve and an independent method of measuring DNA damage levels and repair kinetics are needed in addition to the data in Figure 1A to support the conclusion that there are no differences in repair capacity between the two cell types.

Response: We thank the reviewer for this constructive suggestion to improve our manuscript. As we replied to question #1 and 2, we now examine the formation of 53BP1 foci in PaLung (bat), WI-38 (human), and MEF (mouse) cells after γ-irradiation, as a measure of DNA damage levels and repair kinetics (Fig. 1B). However, given that we performed the assay under only one condition (10 Gy of γ-irradiation) and that there are many other ways to induce DNA damage and repairs, we removed this conclusion to avoid the over-interpreting our data and misleading the readers. We now emphasize that ABCB1-mediated efflux of genotoxins is a unique mechanism in bats to prevent DNA damage, which might contribute to their lower cancer incidence.

6. Many important experiments (Fig 1, Fig 2, Fig 4) are carried out using a single pair of cell lines (WI-38 and PaLung), or compare several bat cell lines only to WI-38. Data for at least one additional human cell line (ideally primary human cells from a different tissue) should be added to these experiments.

Response: We thank the reviewer for the suggestion. We now include additional cell lines derived from human, mouse and bat, to further strengthen our conclusions. In Fig. 2 and the corresponding Supplementary Fig. 2, we added PaKiT03 (bat kidney), PaSpleen (bat spleen), PaBrain (bat brain), IMR90 (primary fibroblasts from human lung), AG01518 (primary fibroblasts from human foreskin), HEK293T (human embryonic kidney cells), and MEF (mouse embryonic fibroblasts) cells. In Fig. 1, 3, and 4, and the corresponding Supplementary

Fig. 1 and 3, we added PaKiT03, HEK293T and MEF cells. In Fig. 7H, we have a total of 12 human cell lines, 2 mouse cell lines, 5 *Pteropus alecto*-derived cell lines, and 4 cell lines from other bat species. Description of each cell line is shown in the Supplementary Table 3.

7. The absolute doxorubicin levels in Fig. 3D should be made available in a format analogous to that in Fig. 2A so the reader can assess the differences among bat, mouse and human cell lines in the absence of the verapamil.

Response: We appreciate this valuable suggestion. We agree with the reviewer that the absolute amount of doxorubicin would be useful to assess the differences between the cell lines. However, unfortunately, there is no method to draw a standard curve to determine the actual amount of intracellular doxorubicin based on doxorubicin fluorescent intensity. The reason we showed relative value of doxorubicin fluorescence in the previous Fig. 3D, instead of showing mean fluorescent value as in the previous Fig. 2A, is because not all the cells were analysed by flow cytometry at the same day. Fluorescent intensity varies from day to day depending on the laser setting/condition of flow cytometry on each day. Therefore, using the raw value of fluorescent intensity does not provide an accurate comparison when data was collected under a different setting or on a different day. We couldn't perform a whole set of experiments in the previous Fig. 3D because it was difficult to obtain the primary bat cells from different tissues and species at the same time, and also because the number of cell lines to conduct was too large to complete all in one day. For the experiment in the previous Fig. 3D, we prepared a pair of cells (i.e. with or without verapamil treatment) at the same time for each cell line and performed flow cytometry assays. Therefore, we think that the comparison of the doxorubicin fold change after verapamil treatment is more reliable to evaluate the ABC transporter-dependent drug efflux, and decided to keep the original format of figure in new Fig. 7H. To further strengthen our conclusion of previous Fig. 2A, we added MEF (Fig. 2), HEK293T and PaKiT03 cells (Supplementary Fig. 2).

8. A positive control for detection of human ABCB1 with the antibody used in the immunoblot presented in Fig. 7 is needed to confirm the absence (or low levels) of the protein in human tissues.

Response: We thank the reviewer for pointing this out. We have now provided a positive control for human ABCB1 detection by including a lysate extracted from HEK293T cells transiently overexpressing human ABCB1 via plasmid transfection (Fig. 7D).

9. The manuscript could be greatly strengthened by an in vivo confirmation of the findings. Although the authors argue persuasively that longevity studies are not feasible with bats, an important test of the role of the ABCB1 transporter in tumorigenesis and tolerance of lethal viruses (Fig. 8) seems feasible by combining ABCB1 inhibition with the relevant exposures. An alternate, simpler in vivo test of the conclusion that bats uniquely resist DNA damaging agents by efflux mechanisms would be to measure LC50 values in bats and mice for a small molecule DNA damaging agent that is an ABCB1 substrate (e.g. doxorubicin) versus an efflux-independent agent (e.g. ionizing radiation).

Response: We would like to thank the reviewer for understanding the difficulties of studying longevity in bats. We fully agree with the reviewer that an *in vivo* confirmation would greatly strengthen our findings in this manuscript. However, due to the strict animal protection laws in

Singapore, live bat experiments are greatly restricted in our area. Hence, we sincerely apologize for our inability to fulfil the reviewer's request, and we hope that he/she will understand.

In addition, as suggested by Reviewer #3, we rewrote our manuscript to focus on the discovery of a unique mechanism for DNA damage resistance via ABCB1 in bats. Therefore, we decided to remove the discussion of ABCB1 in tolerance of viruses.

Yong, K. S. M., et al. Bat-mouse bone marrow chimera: a novel animal model for dissecting the uniqueness of the bat immune system. Sci Rep ***8****, 4726 (2018).*

Reviewer #3

Koh et al., set out to determine if bats cells have better DNA damage response mechanisms than other mammals, which may underlie bats' unexpected longevity. This paper is an elegant, exquisite and in-depth exploration into the response of bat cells to genotoxic chemicals and shows that bats would be excellent models for cancer therapy studies. The exciting finding is that bat cells are able to remove genotoxic substances much faster than human or mouse cells through the ABCB1 transporters. Maybe this could explain the fact that cancer is almost never recorded in bats, not mentioned by the authors.

The authors are correct, this is one of the first studies of its kind, only possible because of the previous cell and genomic tools the authors have developed for bats. However, the authors have oversold or misrepresented this study as a longevity study. It is not a longevity study as such. This is not where its novelty lies. This has lead to a lot of speculation and attempts to tie in bat immunity and potential adaptation to flight (particularly in the discussion), which perhaps is warranted but the data presented here do not show or support these speculations.

I recommend that this paper be resubmitted for another review, once rewritten with a different and more appropriate focus. While the mechanistic studies are exquisite and an example of what is required to establish novel model study species, the ecological and evolutionary aspects of bats presented in this paper are based on old literature that needs updating, sometimes is incorrect, misleading and needs a major rewrite. The results reported are important findings but they need to be correctly repackaged to reach their full impact and not mislead readers. Below are some of my major recommendations aimed to improve this paper and ensure that the bat biology, phylogenetic implications and interpretations are equally as excellent as these novel mechanistic studies.

Response: We really appreciate the reviewer's comments that our paper is an elegant, exquisite and in-depth exploration, and that the results reported here are important findings. We would also like express our gratitude to the reviewer for the valuable suggestions to improve our manuscript. As recommended by the reviewer, we have rewritten the manuscript to focus on DNA damage resistance in bats as a possible tumour suppressive mechanism. We also corrected and updated the information regarding the previous bat studies. To state our responses clearly, we numbered the points raised by the reviewer, below.

1. Page 3, Issues with bat aging data: First line. The authors indicate that the age range for bats is 10-40 years. Certain bat species live longer than 40 years. Myotis brandtii, with the oldest longevity record was at least 41 when re-caught, first captured as an adult of unknown age, considered at least >1 years of age given the fused finger bones, and re-caught 40 years later.

They should use more up to date aging data for bat longevities, which would be AnAge < http://genomics.senescence.info/species/>. According to this website which reflects some of the most up to date literature, bats can live approx. 6-41 years, this reflect the range of longevities in bats Molossus molossus (>5.6 years)- Myotis brantii (>41 years). What they have not done is correct for body size, such as carried out in Austad 2010 J. Comp. Path V142, S10-S21 Fig 2. This is what sets bats apart from other species given their body size, not their actual chronological age, showing that Brandts bats can live up to 10 times longer than would be expected given their body size. This is what makes bats an interesting species to explore exceptional longevity and therefore has driven this study. Although they refer to Max Rubner's rate of living theory they really don't explore what this means in terms of making bats outliers. They need to highlight why bats are so unique given their longevity. This is not clear throughout.

Response: We would like to thank the reviewer for the highly valuable information to update our bat data. Given the refocus of the manuscript as suggested by the reviewer, we now do not substantially discuss bat longevity in the Discussion, although we try to highlight the uniqueness of bat longevity in the Introduction (line 70-84).

2. Using this rational Pteropus alecto is not an exceptionally long lived bat, with an longevity quotient of 1.67 as based on the data set of Healy et al 2014 Proc Roy Soc (DOI: 10.1098/rspb.2014.0298) analysed as in Austad 2010 J. Comp. Path V142, S10-S21 Fig 2. Our estimates for the other species are Cynoterus brachyotis 1.43; the average LQ for the genus Myotis is 4.108 (n=21); the average LQ for the genus Rhinolophus is 5.64 (n=2); Myotis brandtii is (8.23), humans (4.3).

Bats still do live longer than humans given their body size but not all species. They need to clarify the LQ for all the bats in their study to enable the readers get an estimate of where in the aging spectrum these species fall. They should also highlight that these data predominately come from field-based studies so may be an underestimation of true longevities, bats most likely live longer.

Response: We appreciate the reviewer for these suggestions. We found these resources very interesting and thank the reviewer for this input. As stated in the reply for question #1, we focus on DNA damage resistance by ABCB1 and do not extensively discuss bat longevity in the revised manuscript. Therefore, we didn't include the detailed data of bat such as LQ in the revision. Instead, we included a table of the maximum lifespan and body mass of the bats used in our study (Supplementary table 2). We also stated in the Introduction that these values may be an underestimate because their lifespan was determined based on field-based studies (line 73-75).

3. Again the rationale for choice of study species must be described. I presume that one of the reasons for choosing these species is that the authors have already developed the appropriate cells lines and genomic data/tools that enable a study of this kind. This is a valid reason for choosing these taxa and this study does represent some of the first in-depth cellular functional studies in bats but it needs to be made clear. Of the ~1300 species of bats why did they choose Pteropus alecto, Myotis davidii etc. This needs more explanation.

Response: Indeed, we chose *Pteropus alecto* because we have access to it and we have conducted whole genome sequencing and genomic analysis (Zhang, G., *et al.* 2013). We also

established multiple cell lines derived from various tissues of *Pteropus alecto* (Crameri, G., *et al*. 2009). Our genomic analyses demonstrated that their DNA damage response pathway has undergone evolutionary positive selection (Zhang, G.*, et al.* 2013). These results motivated us to use *Pteropus alecto* as a model to explore potential mechanisms of DNA damage resistance in bats. To support our findings, we also performed some of the experiments using *Cynopterus brachyotis*, *Myotis muricola*, *Myotis davidii*, and *Rhinolophus lepidus*, which we also have access to. We now explain the reasons for choosing these species in the Discussion (line 330-331).

Crameri, G., et al. Establishment, immortalisation and characterisation of pteropid bat cell lines. PLoS One ***4****, e8266 (2009).*

Zhang, G., et al. Comparative analysis of bat genomes provides insight into the evolution of flight and immunity. Science ***339****, 456-460 (2013).*

4. Page 3 Reference to enzymatic assessment of free radical scavenging as ref 14 is incorrect, this is the field record for Myotis brandtii longevity, rather than ref 31, which I presume they meant to refer too.

Response: We thank the reviewer for pointing out this error. We have made the correction.

5. Fruitvore such be Frugivore throughout.

Response: We thank the reviewer for pointing out this error and have made the correction.

6. Page 4. The authors state definitively that bats and humans are similarly responsive to DNA damage as induced by irradiation (Fig 1 A). Now I'm intrigued why they did not also test mouse cells here? Both human and bats respond in the same manner but bats should respond more like mice given their body size and metabolism, according to the logic used in this paper. The fact that they respond more similar to human indicates that they are responding more like a large long lived mammal rather than a small short lived mammal as would be expected. Again this is why I feel the underlying rationale for using bats as a model species for aging resistance needs to be better explained throughout.

Response: In the revision, we now include mouse embryonic fibroblast (MEF) as a representative mouse cell (Fig. 1, 2, 3A, 3B, 4A, 4B, 7H, and Supplementary Fig. 1B and 2B). We thank the reviewer for making the suggestion to include mouse cells, which we agree strengthen the conclusions of the manuscript.

Since the reviewer suggested earlier that our study is not primarily a longevity study and that we should focus on the novelty of our findings in genotoxic stress resistance, we did not propose bats as a model species for aging resistance in the revised manuscript. Instead, we focus on the discovery that bats have acquired a novel potential mechanism of cancer resistance. As discussed in a review by Seluanov et al, recent progress in the research of long-lived animals has shed light on their unique cancer resistance mechanisms, such as high-molecular-mass hyaluronan secretion in naked mole rats, multiple copy of *p53* in elephants, and concerted cell death in blind mole rats (Seluanov, A., *et al*. 2018). Therefore, elucidating the mechanism of DNA damage resistance uniquely evolved in bats is valuable and could inform possible novel therapeutic strategies in cancer or chemoresistance. In the revised

manuscript, we tried to explain our rationale for using bat as a model species to study novel mechanisms of DNA damage resistance.

*Seluanov, A., Gladyshev, V. N., Vijg, J., Gorbunova, V. Mechanisms of cancer resistance in long-lived mammals. Nat Rev Cancer **18**, 433-441 (2018).*

7. Page 4. One single part to one figure (Fig 1A) and one experiment is not enough to definitively state that bat's DNA damage response is not driving their unique longevities. Either more data need to be provided or this needs to be toned down (see comments below).

Response: We agree with the reviewer. In the original Fig. 1A, we showed that the levels of γH2AX after 10 Gy of γ-irradiation are similar between human and bat cells. To provide further support, we now include additional cell lines from mouse (MEF) (Fig. 1A), human (HEK 293T), and bat (*Pteropus alecto* kidney-derived PaKiT03) (Supplementary Fig. 1A). We also performed 53BP1 immunofluorescence staining after γ-irradiation, which is another widely-used DNA damage marker (Fig. 1B). However, given that we performed the assay under only one condition (10 Gy of γ-irradiation) and that there are many other ways to induce DNA damage and repairs, we removed the previous sentence that "our data refute alterations to the DDR pathway as a plausible mechanism for increased DNA damage resistance" to avoid the over-interpreting our data and misleading the readers. Regardless of the efficiency of the DNA damage response, our data show that ABCB1-mediated efflux is a novel mechanism that protects bat cells from DNA damage.

8. Page 9. The authors have bat cell lines derived from other bat species, why did they only include these species in the drug efflux comparison (Fig 3D, 3E) and not in all of the other studies? I presume that this is because of the limited resources of the material available from other species but it needs to be highlighted. I would have liked to have seen how the different bat species responded to the different DNA damage experiments. I however understand that this may not be possible, but given the different LQs of these species it would be interesting.

Response: We appreciate this suggestion from the reviewer. We added new Western blotting analyses for γH2AX using cell lines derived from *Cynopterus brachyotis*, *Myotis muricola*, and *Rhinolophus lepidus*, treated with doxorubicin alone or together with the ABC transporter inhibitor verapamil (Fig. 7G). Consistent with the results from *Pteropus alecto* (Fig. 4), these bat species also exhibited a doxorubicin dose-dependent induction of γH2AX, which were prevented by histone eviction once efflux activity was inhibited by verapamil. In addition, ABCB1 protein expression was also confirmed in these cell lines (Fig. 7E). We were not able to obtain enough protein from *Myotis davidii* due to limited access to this material.

9. The fact that these diverse bat species are only included in one experiment does not necessarily warrant a conclusion or statement from the authors that these findings are based on a wide phylogenetic study and one of the first of its kind. Yes it is one of the first mechanistic studies of its kind but it is not based on deep phylogenetic representation from bats for all experiments. This needs further clarification.

Response: We thank the reviewer for pointing out this issue. As stated in the reply for question #8, we added Western blotting analyses for additional cell lines from other bat species to support our conclusions (Fig. 7E and 7G). However, we agree that our studies are still not deep phylogenetic studies, so we rephrased these sentences to avoid over-interpretation.

10. The authors are correct in the efflux experiment, they do sample the basal bat divergences but what they are comparing are the response of wild outbred species' recently derived cell lines to experimental mice and over passaged human cells. Are they not concerned the differential response they see in bats compared with humans and mice is nothing more than a wild versus captive/derived response? Lab mice react in completely different fashion to their wild caught sister-species when faced with the same experimental conditions. It would be great to have a non-bat wild derived cell line, something that lives shorter or what is expected for its body size (e.g. shrew, wild mice). I'm not sure that this is a feasible request given how long it would take to establish these resources. The authors should indicate that this could be a problem, likely or unlikely in the manuscript.

Response: We would like to thank the reviewer for raising this point and understanding the limitations of our resources. Unfortunately, we were not able to acquire a non-bat wild derived cell line due to the strict animal regulations in Singapore. We now discuss the possible effects of wild or laboratory conditions and the limitations of our studies in the Discussion (line 433-442).

11. What is regulating the expression of the ABCB1 in bats? This is an interesting question. miRNAs have been suggested as a possible regulatory mechanism that may be driving extended longevity in bats (Huang et al , BMC genomics 17 (1), 906) this should be included somewhere in the text.

Response: We would to like to thank the reviewer for the suggestion. We agree that it is interesting to know the mechanisms that regulate ABCB1 expression in bats. We elaborated in the Discussion on several known ABCB1 regulators in human and mouse cells such as transcription factors (c-myc, c-jun, HIF-1 and CtBP1) (Grandjean-Forestier, F., *et al*. 2009), miRNAs (Zhu, H., *et al*. 2008; Yang, T., *et al*. 2013), environmental factors (Grandjean-Forestier, F., *et al*. 2009), and virus-related proteins (Hayashi, K., *et al*. 2005; Li, S., *et al*. 2017) (line 365-378). We are currently investigating what transcription factors are responsible for bat *ABCB1* expression based on its promoter sequences. As the reviewer suggested, we also included a discussion about possible miRNA-mediated mechanism for tumour suppression and longevity in bats in the Discussion (Huang, Z., *et al*. 2016) (line 418-426).

Grandjean-Forestier, F., Stenger C., Robert J., Verdier M., Ratinaud M. The P-Glycoprotein 170: Just a Multidrug Resistance Protein or a Protean Molecule? In: ABC Transporters and Multidrug Resistance (eds Wang B, Boumendjel A, Boutonnat J, Robert J). John Wiley and Sons (2009).

Hayashi, K., et al. HIV-Tat protein induces P-glycoprotein expression in brain microvascular endothelial cells. J Neurochem ***93****, 1231-1241 (2005).*

Huang, Z., Jebb, D., Teeling, E. C. Blood miRNomes and transcriptomes reveal novel longevity mechanisms in the long-lived bat, Myotis myotis. BMC Genomics ***17****, 906 (2016).*

Li, S., et al. LHBs can elevate the expression of MDR1 through HIF-1alpha in patients with CHB infection: a comparative proteomic study. Oncotarget ***8****, 4549-4562 (2017).*

Yang, T., et al. MiR-223 modulates multidrug resistance via downregulation of ABCB1 in hepatocellular carcinoma cells. Exp Biol Med (Maywood) ***238****, 1024-1032 (2013).*

Zhu, H., et al. Role of MicroRNA miR-27a and miR-451 in the regulation of MDR1/P-glycoprotein expression in human cancer cells. Biochem Pharmacol ***76****, 582-588 (2008).*

12. Discussion. The authors cannot state that based on their experiments that they refute the DDR pathway as a plausible mechanism for increased damage resistance. This is a little misleading and overarching as they only do one experiment on one bat species (Fig1A). They also seem to have a different interpretation of the findings of Podlutsky et al ref 31. The fact that bat cells respond like primate cells and not like mice cells is unexpected and shows that perhaps DDR in bats is unique given their body size. Also according to Podlutsky et al ref 31, humans have better DDR than mice, as do bats for BER, but not NER pathways. This needs further exploration.

Response: We thank the reviewer for this suggestion. As mentioned in our response to question #7, we have added new data, including an analysis of MEFs. Based on our immunofluorescence staining of 53BP1, we did not observe significant differences in DNA double strand breaks (DSBs) and its repair kinetics between WI-38 and PaLung cells after 10 Gy of γ-irradiation, whereas MEFs were slower in responding to and repairing DNA damage compared to WI-38 and PaLung cells (Fig. 1B). High doses of γ-irradiation, such as 10 Gy used in this study, mainly cause DSBs (Kavanagh, J. N., *et al*. 2013). DSBs are the most detrimental form of DNA breaks for cells and most DSBs are repaired via non-homologous end-joining (Mao, Z.*, et al.* 2008; Kakarougkas, A. and Jeggo, P. A. 2014). 53BP1 is recruited to damaged DNA to facilitate non-homologous end-joining repair and is released upon repair (Panier, S. and Boulton, S. J. 2014; Croco, E.*, et al.* 2017). Our results suggest that the DDR for double strand breaks might be better in humans and bats than in mice, perhaps similar to base excision repair (BER) that was observed by Podlutsky et al as the reviewer mentions.

We agree with the reviewer that our limited results do not exclude that alterations to the DDR pathway contribute to the increased DNA damage resistance. In the revised manuscript, as the reviewer suggested, we deleted these statements to avoid misleading the reader and over-interpreting our results. We also discuss the results from Podlutsky's group regarding the DNA damage repair pathways in different species (Podlutsky, A., *et al*. 2008) (line 390-396).

Croco, E., et al. DNA Damage Detection by 53BP1: Relationship to Species Longevity. J Gerontol A Biol Sci Med Sci ***72****, 763-770 (2017).*

Kakarougkas, A., Jeggo, P. A. DNA DSB repair pathway choice: an orchestrated handover mechanism. Br J Radiol ***87****, 20130685 (2014).*

Kavanagh, J. N., Redmond, K. M., Schettino, G., Prise, K. M. DNA double strand break repair: a radiation perspective. Antioxid Redox Signal ***18****, 2458-2472 (2013).*

Mao, Z., Bozzella, M., Seluanov, A., Gorbunova, V. Comparison of nonhomologous end joining and homologous recombination in human cells. DNA Repair (Amst) 7, 1765-1771 (2008).

Panier, S., Boulton S. J. Double-strand break repair: 53BP1 comes into focus. Nat Rev Mol Cell Biol ***15****, 7 (2014).*

Podlutsky, A., Podlutskaya, N., Bakri, I., Csiszar, A., Ungvari, Z., Austad, S. Comparative analysis of DNA repair pathways in mammals. FASEB J ***22****, 1239-1232 (2008).*

13. Discussion. They do not study multiple phylogenetically distant bats for all experiment just one. Again this is misleading.

Response: We thank the reviewer for pointing out this issue. As mentioned in the response to questions #8 and #9, we added additional data from cell lines derived from other bats to support our conclusions (Fig. 7E and 7G). However, as the reviewer indicated, we couldn't perform a full set of experiments using these phylogenetically distant bat cell lines due to limited access to the materials. Therefore, we rephrased these sentences to avoid over-interpreting the data.

14. Discussion. Why would bats be faced with more vulnerability to a wide spectrum on xenobiotics? Assuming, given the claims the authors make, that this adaptation of removing genotoxic substances is ancestral, the bat ancestor would not have been faced with such a large range of different environments. Bats diverged into their different biological niches over millions of years (Teeling et al, Science 307 (5709), 580-584). This rationale either needs to be removed or better qualified.

Response: We thank the reviewer for raising this point. We agree that our rationale in the previous manuscript was a naive assumption. As the reviewer pointed out, there are no clear implications in the literature that bat ancestors were exposed to more genotoxic substances compared to other mammals such as mice. Therefore, we removed the sentence from the Discussion.

All mammals in wild environments are constantly exposed to varying levels of genotoxic substances, and we speculate that ABCB1 contributes to detoxifying these substances. Consistent with this idea, ABCB1 is specifically expressed in regions of detoxification and protective barriers in most of mammals, including human. In fact, *ABCB1* knockout mice are highly sensitive to genotoxic substances (Schinkel, A. H., *et al*. 1994; Schinkel, A. H., *et al.*1997). Although *ABCB1* knockout mice are not cancer prone, this may be due to laboratory conditions, where the environment and diet are free from genotoxic materials. *ABCB1* knockout mice could be cancer prone in wild conditions. These are now discussed in the Discussion (line 339-349 and line 429-432).

As stated in our reply to question #6, recent findings have revealed unique tumour suppression mechanisms that have evolved in several long-lived mammals. Our data implicate that the high and broad expression of ABCB1 could be a unique tumour suppression mechanism specifically evolved in bats, perhaps reflecting the need to efflux xenobiotic or even intercellular cytokines or metabolic by-products from cells, in order to maintain cellular homeostasis. We elaborated on how bats might have acquired the high and broad expression of ABCB1 in the Discussion (line 358-364 and line 373-378).

Schinkel, A. H., et al. Disruption of the mouse mdr1a P-glycoprotein gene leads to a deficiency in the blood-brain barrier and to increased sensitivity to drugs. Cell 77, 491-502 (1994).

Schinkel, A. H., et al. Normal viability and altered pharmacokinetics in mice lacking mdr1-type (drug-transporting) P-glycoproteins. Proc Natl Acad Sci U S A ***94****, 4028-4033 (1997).*

15. Discussion. Bats don't experience the majority of these toxic substances in the wild. The authors refer to cadmium as a naturally occurring substance. How often to bats experience this toxin? Again I'm not sure what selective pressure would have driven this adaptation in the bat ancestor? The evolution of the ABCB1 expression should predate the massive expansion into all these niches, a pre-adaptation which has allowed bats to expand? But then why did it evolve

in the first place? Again the authors must reconsider and further explore this rationale.

Response: We agree with the reviewer that our rationale was naive. We deleted the sentences regarding this issue and included another possible explanation as to why bat cells have evolved high ABCB1 expression, as mentioned in the response to the question #14.

16. Discussion. The authors need to update their references and value pertaining to the metabolic consequences of flight in bats. There have been many more recent studies on this area.

Response: We thank the reviewer for the recommendation. Since we do not discuss about flight in bats in the revised manuscript, we have done the necessary amendments accordingly.

17. Discussion: The authors cannot state that they have shown in their genome paper (40) that bats have adaptively lost PYHINS to reduce inflammation as an adaptation to flight. This is a gross over statement of what their paper showed, which was simply a lack of PYHINS in bats compared with other mammals which they correlated with flight acquisition. This really is misleading. Also the tenuous link between ABCB1 and anti-inflammation/immunity mechanisms needs to be clarified. It might be correct but there are no data to show this here. I think reporting their new findings in light of these other speculated adaptations is a little contrived. I'm not sure that this brings anything more to this paper and may even dilute the results.

Response: We appreciate the reviewer's advice. We deleted the statements related to inflammation and immunity as well as PYHINS. In the revised manuscript, we now focus on DNA damage resistance by ABCB1 in bats as a possible tumour suppressive mechanism.

18. Discussion. I really commend them on their explanations about why it is so difficult to do any in vivo studies on bats. This is important and further highlights the utility of their system.

Response: We sincerely thank the reviewer for understanding and acknowledging the difficulties we face when exploring and studying unconventional model organisms such as bats.

19. Discussion. Their final statement again is really speculative, Fig 8 again is suggestive. The data don't show this. They need to refer to their real results- bats are better able to remove genotoxic substances from their cells better than mice and humans and this indicates that they could be a great model to study anti-cancer mechanisms. Anything else is speculation and if they want to include it then they need to specify that this is just speculative and suggestive.

Response: We truly appreciate the reviewer's advice. We removed the speculations and drew a new model which is focused on DNA damage resistance via ABCB1-mediated removal of genotoxic substances as a potential tumour suppressive mechanism in bats (Fig. 8).

20. They need to update the literature on aging studies and genomic studies in bats (e.g. Siem et al. Nature Communications 2013, Myotis brandtii genome) and other outlier species naked mole rat, not only self-citing. Some key literature are hi

Response: We thank the reviewer for pointing this out. We have revised the manuscript accordingly and cited the paper by Siem et al. in the Discussion and key literatures of anti-

cancer strategies of long-lived mammals such as a naked mole rat, blind mole rat, bowhead whale, and elephant in the Introduction. However, since we decided to focus on the removal of genotoxic substances by ABCB1 as a potential tumour suppressive mechanism, we have removed some of the references which were not related to this topic.

REVIEWERS' COMMENTS:

Reviewer #1 (Remarks to the Author):

It was good idea to rewrite the manuscript to focus to DNA damage resistance via efflux of genotoxic substances as a potentially novel tumor suppressive mechanism in bats. The manuscript was well written.
One minor point:
The authors may want to cite the original work (Proc. Natl. Acad. Sci. USA 84, 3004-3008, 1987), which showed that human ABCB1 confers multidrug resistance for the first time, for the following sentence "ABCB1 was originally discovered in cancer cells where its high and functional expression promotes resistance to a variety of chemotherapeutic drugs, such as paclitaxel, vinblastine, etoposide and doxorubicin (32, 34)." (lines 350-352)

Reviewer #2 (Remarks to the Author):

I appreciate the thorough, thoughtful responses of the authors to each of my comments. I am satisfied that all of the experiments that can address them have been carried out, or a reasonable explanation has been given as to why they are not feasible.

Reviewer #3 (Remarks to the Author):

The authors have taken on board the majority of my comments. The paper is now streamlined with a more appropriate focus.

There are minor grammar and typographical errors throughout that will need to be corrected.

This is an exciting piece of novel research that will be the basis for future investigations.

Point-by Point response to the reviewers' comments

Reviewer #1 (Remarks to the Author)

It was good idea to rewrite the manuscript to focus to DNA damage resistance via efflux of genotoxic substances as a potentially novel tumor suppressive mechanism in bats. The manuscript was well written.

We would like to sincerely thank the reviewer for this comment.

One minor point:

The authors may want to cite the original work (Proc. Natl. Acad. Sci. USA 84, 3004-3008, 1987), which showed that human ABCB1 confers multidrug resistance for the first time, for the following sentence "ABCB1 was originally discovered in cancer cells where its high and functional expression promotes resistance to a variety of chemotherapeutic drugs, such as paclitaxel, vinblastine, etoposide and doxorubicin (32, 34)." (lines 350-352)

We have noted the reviewer's excellent recommendation and added the citation accordingly in the manuscript (Reference number 40).

Reviewer #2 (Remarks to the Author)

I appreciate the thorough, thoughtful responses of the authors to each of my comments. I am satisfied that all of the experiments that can address them have been carried out, or a reasonable explanation has been given as to why they are not feasible.

We are very delighted by the respond from the reviewer and would like to sincerely thank the reviewer for this comment.

Reviewer #3 (Remarks to the Author)

The authors have taken on board the majority of my comments. The paper is now streamlined with a more appropriate focus.

There are minor grammar and typographical errors throughout that will need to be corrected.

This is an exciting piece of novel research that will be the basis for future investigations.

We sincerely appreciated the reviewer's comment. We too hope our work will be the basis for future investigations.

We have noted the advice from the reviewer and proofread the manuscript. We amended the grammatical and typographical errors accordingly in all the sections of the manuscript (Introduction, Results, Discussion, Methods and Figure legend).
